# A suite of mathematical solutions to describe ternary complex formation and their application to targeted protein degradation by heterobifunctional ligands

**DOI:** 10.1074/jbc.RA120.014715

**Published:** 2020-08-28

**Authors:** Bomie Han

**Affiliations:** Department of Molecular Pharmacology, Discovery Chemistry Research and Technologies, Lilly Research Laboratories, Eli Lilly and Co., Indianapolis, Indiana, USA

**Keywords:** targeted protein degradation, ternary complex, PROTAC, cooperativity, protein degradation, mathematical modeling, molecular pharmacology, kinetics, E3 ubiquitin ligase, drug development, chemical biology, computational biology, heterobifunctional ligand

## Abstract

Small molecule–induced targeted protein degradation by heterobifunctional ligands or molecular glues represents a new modality in drug development, allowing development of therapeutic agents for targets previously considered undruggable. Successful target engagement requires the formation of a ternary complex (TC) when the ligand brings its target protein in contact with an E3 ubiquitin ligase. Unlike traditional drugs, where target engagement can be described by a simple bimolecular equilibrium equation, similar mathematical tools are currently not available to describe TC formation in a universal manner. This current limitation substantially increases the challenges of developing drugs with targeted protein degradation mechanism. In this article, I provide a full, exact, and universal mathematical description of the TC system at equilibrium for the first time. I have also constructed a comprehensive suite of mathematical tools for quantitative measurement of target engagement and equilibrium constants from experimental data. Mechanistic explanations are provided for many common challenges associated with developing this type of therapeutic agent. Insights from these analyses provide testable hypotheses and grant direction to drug development efforts in this promising area. The mathematical and analytical tools described in this article may also have broader applications in other areas of biology and chemistry in which ternary complexes are observed.

One of the reasons conventional drug development approaches fail to yield a therapeutic agent is a lack of expected *in vivo* efficacy despite a high degree of target validation by gene knockout or knockdown. In those cases, it is often concluded that the target protein has a scaffolding function in addition to the enzyme activity that was inhibited by the drug candidate molecule (1). Targeted protein degradation has drawn a lot of attention in recent years partly because of its potential to remove the entire protein and reproduce the gene knockout or knockdown phenotypes.

Targeting a specific protein for degradation is initiated by recruiting the target protein into a ternary complex with an E3 ubiquitin ligase using a ligand that can bind simultaneously to both (2, 3). Once a ternary complex is formed, endogenous E2 ubiquitin ligases transfer the ubiquitin to the target protein in a target-oblivious manner as long as the target protein is oriented in such a way that a surface-exposed lysine side chain is available for ubiquitin conjugation (4). Certain E3 ligases are known to generate a growing chain of ubiquitin conjugation through Lys^48^ of the target-conjugated ubiquitin as an acceptor for addition of another ubiquitin. These Lys^48^-polyubiquitinated proteins are recognized by the cellular proteasome complex and get degraded (5, 6).

There are two different types of ligands that are often utilized for targeted protein degradation: heterobifunctional ligands that are often called PROTAC (proteolysis targeting chimera) (2) and molecular glues (7). A heterobifunctional ligand has a ligand for the target protein connected through a linker to another ligand for an E3 ubiquitin ligase. Because of the modular nature of heterobifunctional ligands, they are often the method of choice. The heterobifunctional ligands can be readily constructed from a collection of existing ligands for the target protein and a list of known ligands for different E3 ligases (8). Small molecule ligands have been characterized for MDM2 (9, 10), IAP (11, 12), CRBN (13, 14), and VHL (15–18). Despite the simplicity in the concept, optimizing the linker type, length, and attachment point to the existing ligand is not a trivial process. All these factors play a role in the overall affinity and efficiency of the ligand for inducing ternary complex formation. Although a large number of the linkers in the literature have a flexible structure in solution, many known X-ray crystal structures of the ternary complex show a tight folding of the linker in such a way to accommodate protein–protein interactions at the interface (19). In this case, the two ligand groups on the heterobifunctional ligand do not act independently of each other. Binding of one end of the ligand to the target protein would cause a large change in the affinity of the other end toward the E3 ligase or vice versa. Such positive or negative cooperativity in two binding events can be critically affected by small changes in the linker length or the structure and by the attachment points of the linker to each of the two ligand groups. Optimizing each of these three components often requires extensive efforts and currently relies largely on empirical outcomes (8). Objective and quantitative understanding of biochemical properties of ligands during the early phase of the development will be very helpful in guiding the direction of the SAR efforts.

A second molecular construct capable of ternary complex formation is known as “molecular glue” (20). Molecular glues are smaller (typically <500 Da) than heterobifunctional ligands (mostly ∼1000 Da) (21) because dual binding functionality is built into a single pharmacophore without the use of a linker, and they typically show no measurable “hook effect” within a large concentration range, unlike the heterobifunctional ligands. Despite their desirable drug-like properties, molecular glues are difficult to identify and even harder to design in a rational manner, even though progress has been made recently in this direction (22). Because they lack a modular structure, it is difficult to predict which E3 ligase is most likely to yield a glue-like ligand for the target protein of interest. As a result, heterobifunctional ligands tend to be the preferred method of choice.

For targeted protein degradation, ternary complex formation can be considered equivalent to target engagement in the traditional sense of drug action. Although ternary complex formation may not guarantee subsequent polyubiquitination and degradation of the target protein (23, 24), none of these would occur without the initial ternary complex. Therefore, screening and characterization of ligands often start with an *in vitro* measurement of ternary complex formation. For traditional drugs that form a binary complex with the target protein, the dose-response curve of the target engagement shows a sigmoidal curve on the semi-log plot, reaching a plateau at sufficiently high ligand concentrations. This behavior is elegantly described by a simple mathematical equation of [B] = [B]_max_ × [L]/([L] + *K_d_*), where [B] is the ligand-bound concentration of the target protein, [B]_max_ is the total target protein concentration, [L] is the free ligand concentration at equilibrium, and *K_d_* is the equilibrium dissociation constant of the protein–ligand binary complex. In this binary complex system, two easily measured parameters, [B]/[B]_max_ and *K_d_*, have good predictive value for drug efficacy and potency, respectively. The higher fractional target occupancy, the higher biological response to drug, or efficacy, is expected. The lower the *K_d_* value is, the drug is expected to be more potent or elicit the same biological responses at lower drug concentrations when everything else is equal. In comparison, the dose-response curve of the ternary complex shows the hook effect, or a bell-shaped curve reaching a maximum at certain ligand concentration but falling back down to baseline level at sufficiently high concentrations (12, 25–32). Because of this biphasic response, careful dose titration is required for successful degradation of the target using the heterobifunctional ligands. In addition, there are currently no easily measurable biochemical properties that can address the potential efficacy and potency of these molecules as a drug, adding to the long list of challenges in developing therapeutic agents in this mechanism.

Currently, no mathematical equation is available to describe the hook effect and full equilibrium binding characteristics of the ternary complex in a universal manner despite widespread occurrence of the ternary complex in multiple scientific disciplines (reviewed in Ref. 33). It was even demonstrated that solving an exact algebraic equation for the ternary complex as a function of total ligand concentration is mathematically “unsolvable” when the system has cooperativity (33). Analytical solution could be obtained only for a noncooperative equilibrium system (33). Cooperative interaction among the components within the ternary complex induced by the heterobifunctional ligands or molecular glue ligands, however, is considered a critical component of efficient target engagement or target degradation (19, 34). Lack of proper mathematical and analytical tools to directly address such interaction makes it difficult to relate the experimentally measured data to the equilibrium constants or biochemical properties of the ligand. When the desired outcome of efficient target protein degradation is not achieved, it is difficult to sort out where the problem is and how to fix it. A universal mathematical description of the ternary complex system of all types that can connect the experimentally measured data to the biochemical properties of the complex such as equilibrium constants, potency, and efficacy is sorely desired.

In this article, I provide an exact and universal mathematical description of the ternary complex system at equilibrium and its variations that are commonly found in biological systems. This was made possible by solving the mathematical relationships among different components in terms of free ligand concentration at equilibrium rather than total ligand concentration or the initial ligand concentration. Although free ligand concentration is usually not directly measurable, the binary equilibrium equation of [B] = [B]_max_ × [L]/([L] + *K_d_*) is also written in terms of free ligand concentration at equilibrium. This binary equilibrium equation has been universally adopted by scientists of all fields for many decades, and its impact on pharmacology and drug discovery is immeasurable. A similar mathematical equation for the ternary complex that works universally will be extremely valuable. Using mathematical modeling of the system, mechanistic understandings could be obtained for many commonly encountered challenges during development of reagents for targeted protein degradation. Finally, analytical tools were developed that can extract information on potency and efficacy for target engagement, as well as equilibrium constants from the experimental dose-response data. The suite of mathematical tools provided in this article will be helpful in advancing this exciting field of targeted protein degradation and any other discipline involving a ternary complex.

## Results and discussion

### Exact and universal mathematical equations for the ternary complex system at equilibrium

**Figure 1.**
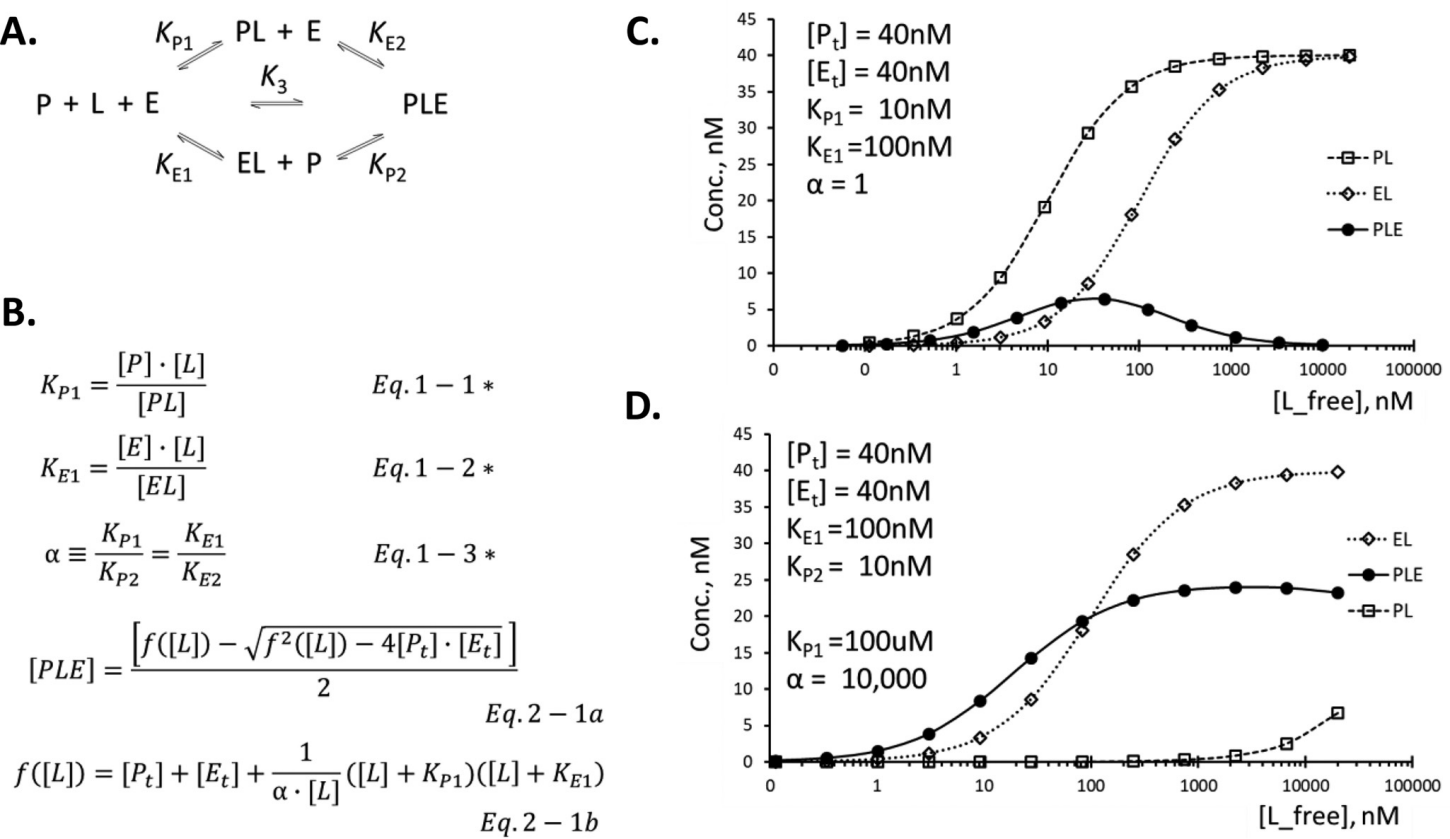
**Mathematical description of a ternary complex at equilibrium.** Binding reactions among target protein (*P*), E3 ligase (*E*), and a ligand (*L*) to form a ternary complex (*PLE*) are shown in *A*. Each of these binary binding events indicated by a pair of *double arrows* are dictated by the dissociation equilibrium constants shown above or below the *arrows*. Because of constraints by the thermodynamic principle of pathway independence, the system can be completely described by the three independent equilibrium constants denoted by *asterisks* shown in *B*. Equilibrium concentration of the ternary complex at a given free ligand concentration is governed by Equations 2 – 1a and 2 – 1b. Definitions of the other equilibrium parameters and a full mathematical description of the system can be found in File S1*A*. In *C*, calculated equilibrium concentrations are shown for the ternary complex, PLE, as well as the two binary complexes in the system, PL and EL, for a typical heterobifunctional ligand under the conditions indicated. A typical situation for molecular glue ligands is depicted in *D*. *Conc*., concentration.

An equilibrium binding of a heterobifunctional ligand (*L*) with its target protein (*P*) and an E3 ligase (*E*) shown in Fig. 1*A* can be completely described by three independent equilibrium constants, *K*_P1_, *K*_E1_, and α, as defined by Equations 1 – 1, 1 – 2, and 1 – 3 in Fig. 1*B*. Note that *K*_P1_ and *K*_E1_ are binary equilibrium dissociation constants, whereas the third parameter, α, is the ratio between the ternary equilibrium dissociation constants (*K*_P2_ and *K*_E2_) and the corresponding binary equilibrium dissociation constants. As such, α is considered a cooperativity factor by many in the field (19, 33). When α is greater than 1, there is a positive cooperativity, whereas a value less than 1 indicates negative cooperativity between the first and second binding events on the same path within the equilibrium diagram. A value of 1 indicates no cooperativity.

Universal mathematical equations for this ternary complex system at equilibrium were solved for the first time and are described in Fig. 1*B*. In short, the concentration of the ternary complex, [PLE], at equilibrium as a function of the free ligand concentration, [L], is given by

$$\left[\mathrm{PLE}\right]=\left[f\left(\left[L\right]\right)-\sqrt{f^{2}\left(\left[L\right]\right)-4\left[P_{t}\right]\cdot \left[E_{t}\right]}\right]/2$$

$$\text{where}\;f\left(\left[L\right]\right)\;=\left[P_{t}\right]+\left[E_{t}\right]+\frac{1}{\alpha \cdot \left[L\right]}\left(\left[L\right]+K_{P\text{1}}\right)\left(\left[L\right]+K_{E\text{1}}\right)$$

where [P_t_] and [E_t_] are total concentrations for the target protein and E3 ligase, respectively. Mathematical equations for the concentration of other species in this diagram and full derivation of these equations are provided in File S1*A*.

Douglas *et al*. (33) have previously described a mathematical equation for a ternary complex system, but their solution was limited to a noncooperative system. They have proven that an analytical solution did not exist for a system with a cooperativity. Considering that most ternary complex systems involving heterobifunctional ligands or molecular glues have high degree of cooperativity, a universal solution for the ternary complex system regardless of the cooperativity was critically needed. The equations in this article were solved with the cooperativity factor α built into the model, and the solutions apply universally to all ternary complex systems regardless of α. The key difference between the two mathematical approaches is that the previous work (33) used total ligand concentration, whereas the current work described the system in terms of the free ligand concentration at equilibrium. An additional benefit of the current work is that these equations are easy to modify to accommodate variations in the system that occur frequently in biological systems as described in detail below.

There is a value in being able to calculate the ternary complex concentration in terms of the total ligand concentration because the free ligand concentration is usually not known because of ligand depletion. Although a universal analytical solution does not exist for the concentration of the ternary complex as a function of the total ligand concentration (33), numeric solution can be easily obtained using the mathematical equations provided in this article. This method is explained in File S1*B*, and a template is provided with step-by-step instructions in an Excel file in supporting information (BHan_PLEcalc_v1.2_200727.xlsx). The familiar equation for the binary complex system, [B] = [B]_max_ × [L]/([L] + *K_d_*), is also written as a function of the free ligand concentration at equilibrium, and use of total ligand concentration in this equation causes overestimation of the concentration of the bound ligand caused by ligand depletion. The numeric method described in this article can be also used for the binary complex system with a simple modification.

The mathematical equations for the two binary complexes in this system, PL and EL, have forms similar to that for a simple binary complex system (Equations 1–8 and 1–10 in File S1*A*), and the saturation binding curves adopt a sigmoidal shape (*open symbols* with *dotted lines* in Fig. 1*C*) in a manner similar to the simple binary complex systems. The equation for the ternary complex, PLE, takes up a more complicated form (Equations 2 – 1a and 2 – 1b in Fig. 1*B*) and produces a symmetrical bell-shaped curve (*filled circles* with a *solid line* in Fig. 1*C*) on the semi-log scale, reproducing the well-known hook effect of the heterobifunctional ligands. Unlike the binary complexes (EL and PL) that reach saturated binding at sufficiently high concentrations of the ligand, the ternary complex (PLE) reaches only a fraction of the total protein concentration even at the height of the bell-shaped curve.

Another well-known class of ligands that induces a ternary complex is molecular glues. They typically do not have any discernable modular structure, and many known molecular glue ligands do not show measurable affinity for the target protein in the absence of the E3 ligase (13, 35–40). The mathematical equations provided in Fig. 1*B* apply equally well to this type of ligand because the mathematical solutions contain no assumptions on the structure or biochemical property of the ligand or the proteins. Low affinity of the ligand for the target protein in the absence of the E3 ligase is captured by a large *K*_P1_ value and a decent *K*_P2_ value, resulting in a high cooperativity factor α. This relationship among equilibrium constants shows up as a broad shape for the ternary complex dose-response curve (*filled circles* with a *solid line* in Fig. 1*D*), mimicking the traditional sigmoidal curve within practical range of the ligand concentrations. At even higher concentrations that are usually not achievable in experimental settings, the concentration of the ternary complex is predicted to fall, following the bell-shaped curve of the heterobifunctional ligands (Fig. 1*C*). For molecular glue ligands in this class, they are mathematically indistinguishable from the heterobifunctional ligands with a very high cooperativity factor.

For extreme cases of molecular glue ligands in which the *K*_P1_ value approaches infinity, the upper path in Fig. 1*A* is effectively blocked, and the system is better described by a series of bimolecular binding events with *K*_E1_ and *K*_P2_ as equilibrium dissociation constants for each step. There will be no need to introduce the cooperativity factor in this case, and the dose-response curve for the ternary complex strictly follows a sigmoidal saturation binding curve of a conventional binary binding system with an overall dissociation equilibrium constant of *K*_PLE_ = *K*_E1_ × *K*_P2_. In the subsequent sections, discussions will focus on traditional heterobifunctional ligands because this is the area in which mathematical understanding is mostly lacking.

### Kinetic simulation of ternary complex formation and independent validation of the mathematical solutions

Each of the mathematical equations provided in Fig. 1*B*, and the method in File S1*B* has been checked for internal consistency by back-calculating equilibrium dissociation constants and total protein concentrations from the calculated concentrations of various species under numerous test conditions (data not shown). To further validate the mathematical solutions in an orthogonal manner, an Excel-based program was developed to simulate the kinetics of binding events in this ternary complex system, and the steady-state concentrations of various species were compared with the concentrations predicted from the analytical solution. Kinetic simulations with a wide range of test parameters tracking real-time progression showed that formation of ternary complex was surprisingly fast, reaching steady-state within 30 min of incubation in most realistic ligand concentrations.

**Figure 2.**
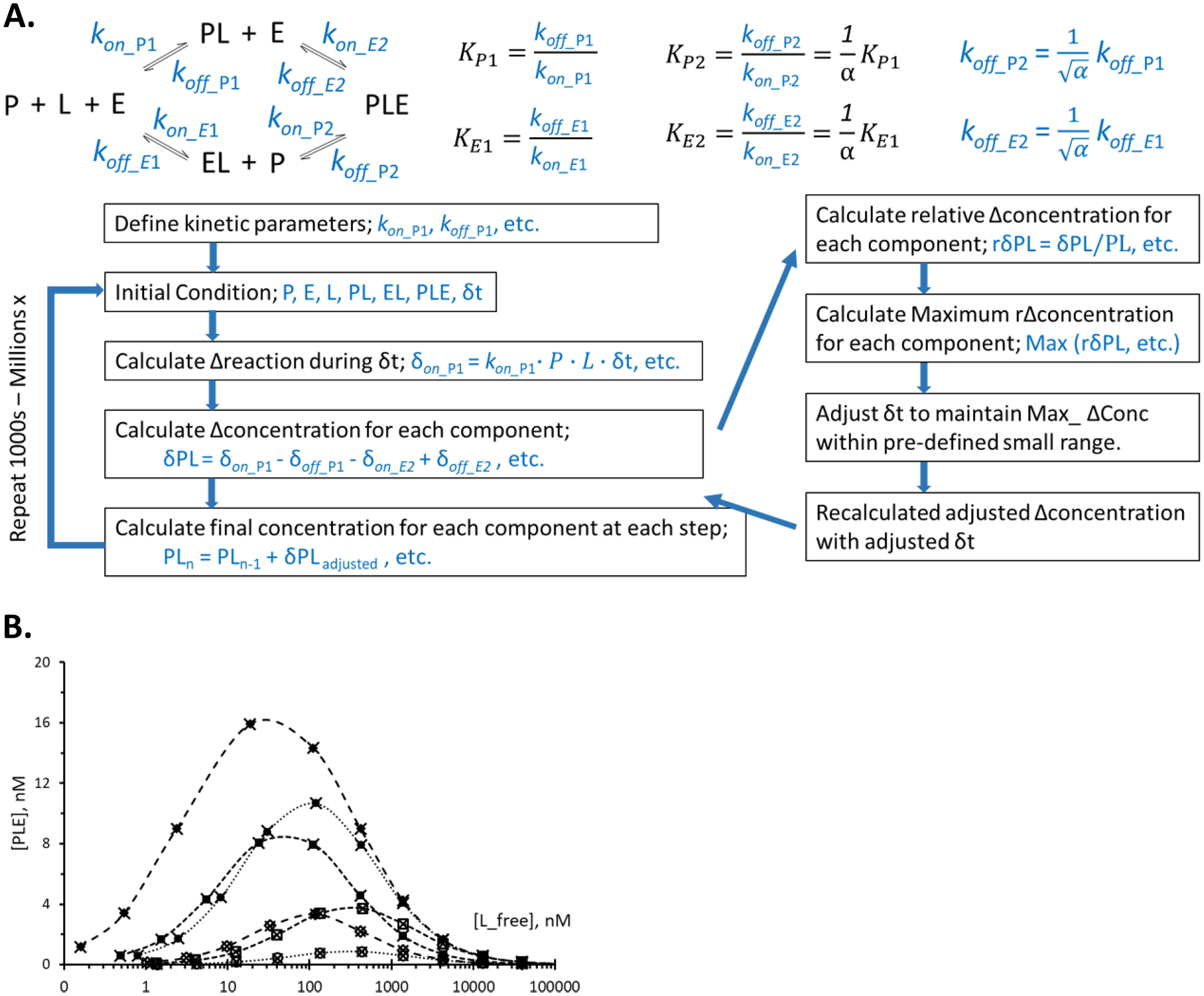
**Kinetic simulation of the ternary complex formation.** The basic algorithm for an Excel-based kinetic simulation program is shown in *A*. The central path in Fig. 1*A*, in which three molecules bind simultaneously in one step, is omitted in this diagram for clarity. Omission of this path in the actual simulation only affects the simulation speed but does not affect the equilibrium outcomes. Additional explanation on the program algorithm is provided in File S2, and a sample program is provided as an Excel file in the supporting information (BHan_TCKinSim_v3.5.4_200505.xlsx). In *B*, concentrations of the ternary complex from the kinetic simulation (shown by *cross-hairs*) are compared with the concentrations calculated by the mathematical equations (Fig. 1*B*) under the same condition (shown by *open* and *filled circles*, *rectangles*, and *diamonds*). Twelve-point dose-response experiments were simulated using six different sets of equilibrium constants representing a wide range of experimental conditions.

Basic algorithm for this kinetic simulation program is given in Fig. 2*A*, with additional descriptions in File S2. Because the program is written in Microsoft Excel, the program can be executed in virtually any personal computer equipped with this basic software. User-provided equilibrium constants and other parameters can be used to fit the system of interest. Six different test conditions were chosen by defining different combinations of *K*_P1_, *K*_E1_, and α for the *in silico* heterobifunctional ligands. For each test condition, simulation was performed with 12 different total ligand concentrations emulating a 12-point dose-response experiment. As a comparison, equilibrium concentrations of the ternary complex were calculated using the method described above. Outcomes of these two approaches are compared in Fig. 2*B*, and the results from the two totally independent methods agreed very well with each other, validating both methods.

The core algorithm for this kinetic simulation program can be applied to any other types of binding reactions, as well as chemical reactions, with minimal modifications of the program. The program described in File S2 can run up to 12 different reaction conditions simultaneously so that a 12-point dose-response experiment can be simulated in one step. This program can be easily adapted to other reaction types and conditions of interest and will be a useful tool beyond simulating the ternary complex system.

### Commonly occurring variations of the ternary complex system with additional equilibria

**Figure 3.**
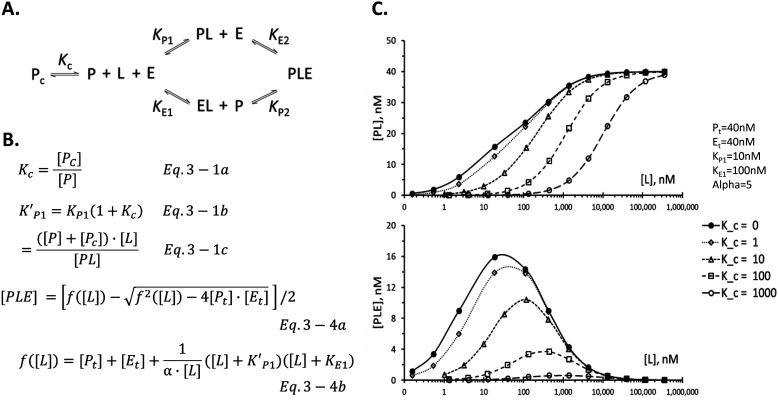
**A common variation of the ternary complex system with an additional equilibrium.** In many biological and biochemical systems, additional equilibrium reactions are connected to the basic equilibrium shown in Fig. 1*A*. One such example is shown in *A*, where the target protein exists in two different forms, *P*_c_ and *P*, with the conformational equilibrium constant *K*_c_ defined in Equation 3 – 1a in *B*. The effect of this additional equilibrium to the mathematical solutions is to replace the *K*_P1_ in Fig. 1*B* with $K_{\mathrm{P1}}$ in Equation 3 – 1b as shown in *B*. Other common variations include the presence of natural ligand that competes with the exogenous heterobifunctional ligand and a coexistence of both of these extra equilibria. Mathematical equations to handle these variations and derivation of all these equations are described in File S3 along with a comparison with their counterparts in the binary complex system. In *C*, the effects of this extra conformational equilibrium of the target protein on the calculated dose-response curves are compared between the binary complex PL (*top panel*) and the ternary complex PLE (*bottom panel*) in the same ternary complex system at equilibrium.

Many proteins exist in multiple conformational states both *in vivo* and *in vitro*, not all of which can bind the desired ligand. Fig. 3*A* describes a ternary complex system with an additional conformational equilibrium of the target protein between closed conformation, *P*_c_, and an open conformation, *P*. Only the protein in open conformation can bind the ligand. The math to describe this system is identical to those in Fig. 1*B* except that the binary equilibrium dissociation constant of the ligand for the target protein, *K*_P1_, is modified in such a way that the apparent dissociation constant, $K_{\mathrm{P1}}$, is given by $K_{\text{P}1}^{\prime }$ = *K*_P1_ × (1 + *K*_c_), where *K*_c_ is the conformational equilibrium constant given in Equation 3 – 1a in Fig. 3*B*. Note that the physical meaning of $K_{\mathrm{P1}}$ is the *K*_P1_ when it is expressed in terms of total unbound target protein concentration (Equation 3 – 1c) rather than concentration of the target protein in the open conformation. Substitute $K_{\mathrm{P1}}$ into *K*_P1_ in Equations 2 – 1a and 2 – 1b in Fig. 1*B* to obtain Equations 3 – 4a and 3 – 4b in Fig. 3*B* to calculate the equilibrium concentration of PLE. Similarly, concentrations of all other components can be obtained by simple substitution of *K*_P1_ with $K_{\mathrm{P1}}$ from equations in File S1*A*. Mathematical derivations of the equations in Fig. 3*B* are provided in File S3 (*part 2A*).

Another frequently encountered case of modified equilibrium involves competition by a monofunctional ligand for the target protein as shown in File S3 (*part 1B*). Many therapeutically interesting proteins have endogenous ligands such as ATP for kinases or GTP for small G-proteins including RAS proteins. Many compounds used for development of the heterobifunctional ligands bind to the same pocket as occupied by these endogenous ligands. Similar to the above case of conformational equilibrium, the equilibrium concentrations of the ternary complex and all other species can be calculated by a simple substitution of the *K*_P1_ with $K_{\mathrm{P1}}$ = *K*_P1_ × (1 + [C]/*K_i_*), where [C] is the concentration of the competitor, and *K_i_* is the equilibrium dissociation constant of the competitor for the target protein P (Equations 3 – 2a and 3 – 2b in File S3, *part 1B*). When monofunctional ligand for the E3 ligase is used, a similar substitution is used for *K*_E1_. Mathematical derivations of the equations for this system are provided in File S3 (*part 2B*). Note the close parallel in the mathematical solutions for a system with competition between the ternary complex (Equations 3 – 2a and 3 – 2b in this article) and the binary complex (File S3, *part 3*), commonly known as the Cheng–Prusoff equation (41).

These two different variations of the system often occur simultaneously in the same target protein in the cellular system. For examples, C-RAF protein has an N-terminal regulatory domain that binds the C-terminal catalytic domain to keep it from binding ATP (42). This intramolecular interaction is further strengthened by binding of another protein, 14-3-3, to two phosphorylated Ser residues, Ser^259^ and Ser^621^, with Ser^259^ being on the N-terminal domain and the Ser^621^ on the C-terminal domain. Simultaneous binding of 14-3-3 to both phosphoserine residues keeps the protein in the closed conformation, safeguarding the C-RAF protein from accidental binding of ATP and activation. Thus, targeting the C-RAF for degradation with heterobifunctional ligands faces challenges from both types of additional equilibria in the ternary complex system if the heterobifunctional ligands are designed to bind to the same ATP-binding pocket. This situation is depicted in File S3 (*part 1C*). In this case, the two extra equilibria contribute additively to the increase in apparent dissociation constant following the equation, $K_{\mathrm{P1}}$ = *K*_P1_ × (1 + *K*_c_ + [C]/*K_i_*) (Equations 3 – 3a, 3 – 3b, and 3 – 3c, in File S3, *part 1C*). Similar to above two cases, equilibrium concentrations for all species in this system can be calculated by substitution of $K_{\mathrm{P1}}$ in place of *K*_P1_. Mathematical derivations of the equations for this system are provided in File S3 (*part 2C*).

Although the mathematical modifications needed to incorporate the extra equilibria into the ternary complex system are very similar to those of the binary complex system (see File S3, *part 3* for comparison), the consequences on the target engagement are very different. For binary complexes, whether they are simple binary complexes or are part of the ternary complex system, the binding curves in the presence of these extra equilibria reach the same maximum level as in the absence at sufficiently high ligand concentrations (Fig. 3*C*, *top panel*). For ternary complexes (Fig. 3*C*, *bottom panel*), the presence of these extra equilibria not only shifts the center of the curve to the *right* but also significantly reduces the maximum level of the ternary complex. As the experimental system moves from *in vitro* biochemical system to the cellular system, there will be both right shift in the efficacious concentration and reduction in the maximal engagement of the target protein into the ternary complex unless these extra equilibria are already incorporated into the *in vitro* biochemical system. From this analysis, it is clear that a ternary complex–forming ligand that binds to an area outside the binding pocket for endogenous ligand has an advantage over ligands that compete with the endogenous ligand. In the same manner, a ligand that binds to all conformations of the target protein has an advantage over ligands that are conformationally selective. Because most efforts to develop heterobifunctional ligands for the purpose of targeted protein degradation start with existing collection of inhibitors for the target protein, it is important to understand the mode of binding of these reagents and choose them carefully to avoid the issues laid out above from the start.

### Understanding the roles of individual equilibrium constant on target engagement: potency and efficacy in inducing ternary complex formation

**Figure 4.**
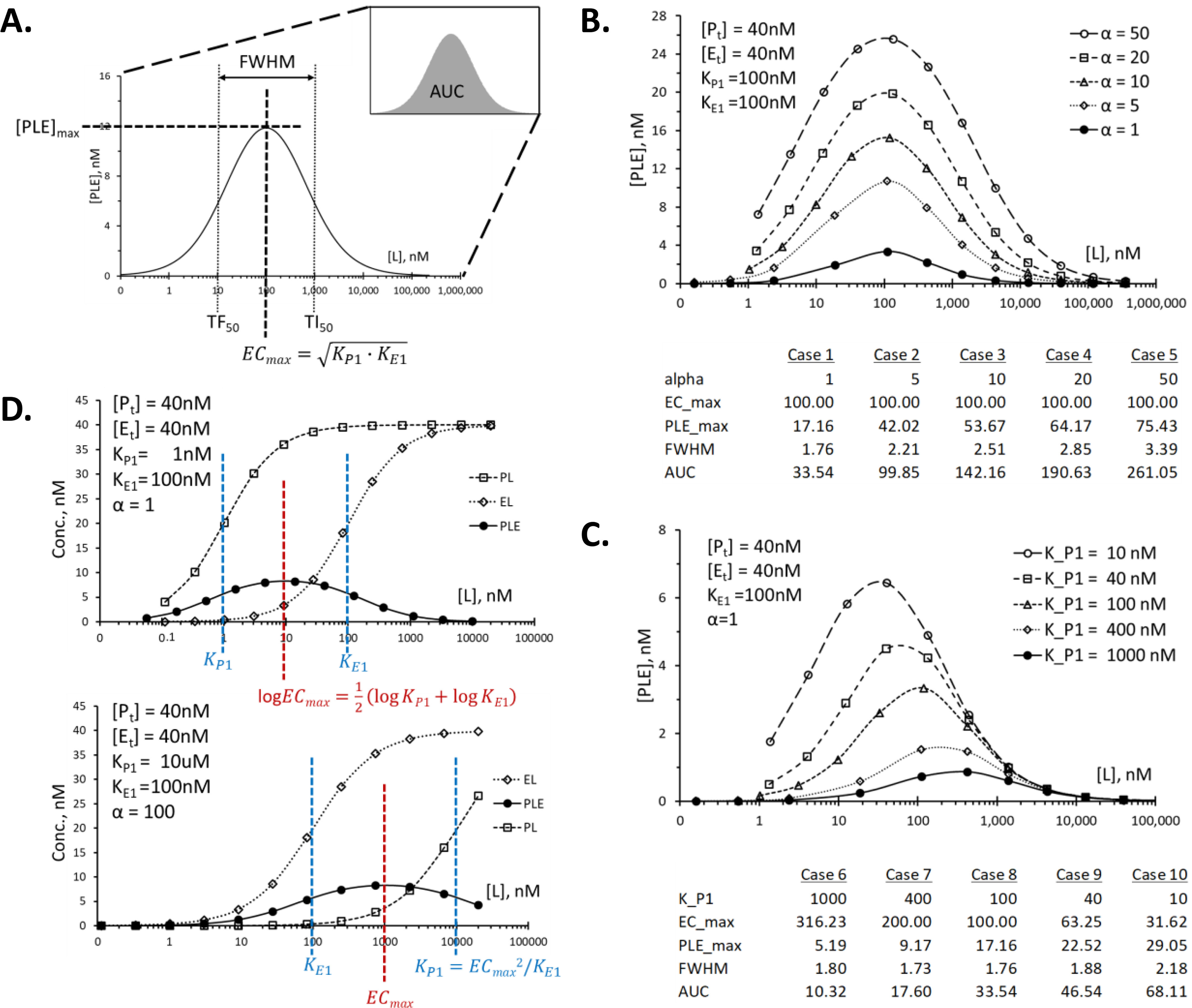
**The role of α and *K*_P1_ on target engagement.** *A* shows geometric properties of the typical dose-response curve that are useful in addressing efficacy and potency of the heterobifunctional ligands. Note that the *center* of the *bell-shaped curve*, named EC_max_ for maximally effective concentration, is the geometric mean of the two binary equilibrium constants, *K*_P1_ and *K*_E1_. [PLE]_max_ is for maximum concentration of PLE. TF_50_ and TI_50_ are for concentration that gives 50% of maximal response on *left* and *right sides* of the *bell-shaped curve*, respectively. The pharmacological meanings of these parameters are explained in the text, and their mathematical equations and derivations are provided in File S4. The increase in cooperativity factor α resulted in increased [PLE]_max_ and a concomitant increase in AUC without changing EC_max_ as shown in *B*. The decrease in *K*_P1_ value or the increase in affinity of L for P resulted in both increase in the [PLE]_max_ and a decrease in EC_max_ value with a simultaneous increase in AUC as shown in *C*. Implications of the mathematical equations for EC_max_ are depicted in *D*. On the semi-log plot of [PLE], the position of the EC_max_ is in the middle of the *K*_P1_ and *K*_E1_. Saturation binding curves for PL and EL are for a simple binary complex system involving only P + L or E + L, respectively. *Conc*., concentration.

For objective and quantitative description of *in vitro* target engagement, the following geometric properties of the bell-shaped ternary complex dose-response curve were defined, and their utility was evaluated (Fig. 4*A*). The first is EC_max_ for maximally effective concentration, which corresponds to the position of the apex of the bell-shaped curve on the semi-log plot. Derivation of the mathematical equation for EC_max_ ($=\sqrt{K_{\text{P}1}\cdot K_{\text{E}1}}$) can be found in File S4 (*part 2A*). The second feature is [PLE]_max_ for maximum concentration of the ternary complex PLE obtained when the ligand concentration is the same as EC_max_. Because of the biphasic nature of the dose-response curve, it may be useful to define the range of concentrations that gives at least half-maximal response or FWHM for full width at half-maximal points. On the semi-log plot, this corresponds to the distance between two EC_50_ values, the first of which is defined as TF_50_ for ternary complex–forming EC_50_ and the second TI_50_ for ternary complex–inhibitory EC_50_ according to the convention introduced earlier (33). The last item is AUC for area under the curve, which can be numerically calculated by dividing the horizontal axis into small segments and adding up the areas of all the small rectangles using the mathematical solution for [PLE] as the height of the individual rectangle. Among many geometric properties of the dose-response curve, AUC can be the most useful parameter to address the efficacy, or the overall effectiveness, of the heterobifunctional ligand because it combines the range of effective concentrations and extent of target engagement across all concentrations into a single value. In comparison, EC_max_ addresses the potency of the ligand by indicating the most effective concentration of the ligand for maximal target engagement. Mathematical equations for each of these parameters are provided in File S4 (*part 1*), and their derivations are shown in File S4 (*part 2*). The mathematical equation for AUC is not available, but an alternative solution for AUC will be discussed later.

Using the equations for the ternary complex shown in Fig. 1*B* and File S4 (*part 1*), contributions of different equilibrium constants on the above-defined geometric properties of the dose-response curve were evaluated. When cooperativity factor α was increased from 1 to 50 while the two equilibrium dissociation constants, *K*_P1_ and *K*_E1_, were kept constant, there was a progressive increase in [PLE]_max_ without affecting the EC_max_ (Fig. 4*B*). Both FWHM and AUC increased with an increasing α value. In Fig. 4*C*, the *K*_P1_ value was changed progressively from 1 μm to 10 nm, whereas the cooperativity factor α was kept constant. Both EC_max_ and [PLE]_max_ values showed a consistent change toward lower EC_max_ and higher [PLE]_max_. Changes in *K*_E1_ value produced the same effect (data not shown). Interestingly, although AUC increased with decreasing *K*_P1_ value, FWHM showed an inconsistent pattern. From these examples and others, an empirical formula of AUC ≅ 1.1 × FWHM × [PLE]_max_ was found.

From these analyses, it was discovered that EC_max_ is determined solely by the combined effect of binary equilibrium constants, *K*_P1_ and *K*_E1_, and not affected by the cooperativity of the system or the total concentrations of the proteins used. For this reason, EC_max_ can be regarded as a unique equilibrium parameter for a given TC-forming ligand independent of the specific assay condition. The mathematical equation for EC_max_ ($=\sqrt{K_{\text{P}1}\cdot K_{\text{E}1}}$) supports this notion. EC_max_ value provides a measure of potency in the ternary complex system, and it may be regarded as an equivalent of EC_50_ in the binary complex system with one very important distinction. In the binary complex system, all concentrations higher than EC_50_ value gives higher concentrations of the binary complex. In the ternary complex system, however, concentrations both lower and higher than EC_max_ give less amount of the ternary complex. Both [PLE]_max_ and AUC showed a consistent increase with either increase in cooperativity factor or with increase in binary affinity of the ligand for the target protein or the E3 ligase. For this reason, both parameters will be useful in describing overall effectiveness, or the efficacy, of the ligand in inducing ternary complex formation. On the other hand, FWHM did not show a consistent trend. Therefore, FWHM itself may not be used as a parameter to compare efficacy across different ligands, whereas it may be a useful parameter in defining the dynamic range of a given ligand in combination with EC_max_.

It is important to understand that, except for EC_max_, all other parameters described above are dependent on the total concentration of the proteins, [P_t_] and [E_t_], as well as the three equilibrium constants, *K*_P1_, *K*_E1_, and α (see equations in File S4, *part 1*). Therefore, when using these parameters to compare different heterobifunctional ligands, the assay condition needs to be specified and fixed for proper comparison. When these geometric parameters were compared for the same ligand between two assays using different total protein concentrations, normalization by total protein concentration removed most (80–95%) of the variation (data not shown), but an error-free method of normalization could not be found.

One very useful property of EC_max_ emerges when its relationships with *K*_P1_ and *K*_E1_ are examined graphically (Fig. 4*D*). Because the value of EC_max_ is the geometric mean of *K*_P1_ and *K*_E1_, the position of the EC_max_ on the semi-log plot is in the middle of the two binary equilibrium dissociation constants, as illustrated in Fig. 4*D* (*top panel*). This understanding provides an insight into one of the frequently encountered problems during early phase of SAR for the development of the heterobifunctional ligands. Often, the affinity of the heterobifunctional ligand for the target protein is so low that it is difficult to obtain a full saturation curve in a binary binding experiment as depicted in Fig. 4*D* (*bottom panel*, *open square symbols*). Even with these seemingly very poor ligands, it is not uncommon to obtain a well-defined bell-shaped binding curve for the ternary complex especially if the cooperativity factor is sufficiently high. Without mathematical understanding of the system, one might prematurely conclude that these two sets of data are inconsistent with each other and that one of these measurements is not working properly. Understanding the underlying mathematical relationships helps resolve these conflicts. The *K*_P1_ value can be estimated from the relationship, *K*_P1_ = EC_max_^2^/*K*_E1_, if the *K*_E1_ value is known.

During SAR of heterobifunctional ligands, it is often observed that simple changes in the linker length lead to drastic changes in the effectiveness of the compounds in degrading the target proteins (34). This can be most readily explained by changes in the cooperativity because optimal protein–protein interaction between the target protein and the E3 ligase requires a certain length of the linker to allow favorable interaction of the two proteins without clashing (19). Care must be taken to confirm this interpretation, because the binary binding affinity can and does change with different linker lengths (34). The change in [PLE]_max_ or AUC without change in EC_max_ will be a diagnostic test for change in cooperativity without change in binary affinity. Focusing on binary binding affinity (*K*_P1_ or *K*_E1_) or the cooperativity (α) without regard to the other may lead to erroneous conclusion on the cause of differences in target degradation. Overall target engagement captured by AUC may provide a better explanation for differences in target degradation. It is also important to keep in mind that not all target engagement may be productive. The concept of a “lysine desert” (43) has been proposed to explain stability of certain proteins against proteasome-mediated degradation. Plasticity in binding has been shown to play a key role in selectivity in ligand-induced protein degradation among related proteins (24). Depending on the proximity and orientation of the target protein in the ternary complex relative to the donor ubiquitin, ternary complexes may be classified as productive *versus* nonproductive. In this light, some ligands may act as “agonist” for certain target proteins, whereas the same ligands may act as “antagonist” for other proteins in the same family. Proper comparison of “agonism” across different ligands or “productivity” of different ternary complexes should be based on the equivalent level of target engagement rather than any of the individual equilibrium constants.

One of the major challenges in developing heterobifunctional ligands as a therapeutic agent is a low exposure level of these agents because of their sizes and limitation on solubility. One obvious way to overcome this challenge is to increase the affinity of the heterobifunctional ligand for either the target protein or the E3 ligase, or both, because the ligand will become more effective in inducing ternary complex formation at low concentrations as shown in Fig. 4*C*. Considering the large right shift of the EC_max_ and the reduction in [PLE]_max_ by the common occurrence of extra equilibria for the target proteins as described in Fig. 3*C*, it is hard to overemphasize the importance of achieving highest affinity of the heterobifunctional ligand for both target protein and the E3 ligase to compensate for the loss of target engagement by the presence of extra equilibria.

### Quantitating target engagement from experimentally measured ternary complex dose-response data: Gaussian curve fitting

**Figure 5.**
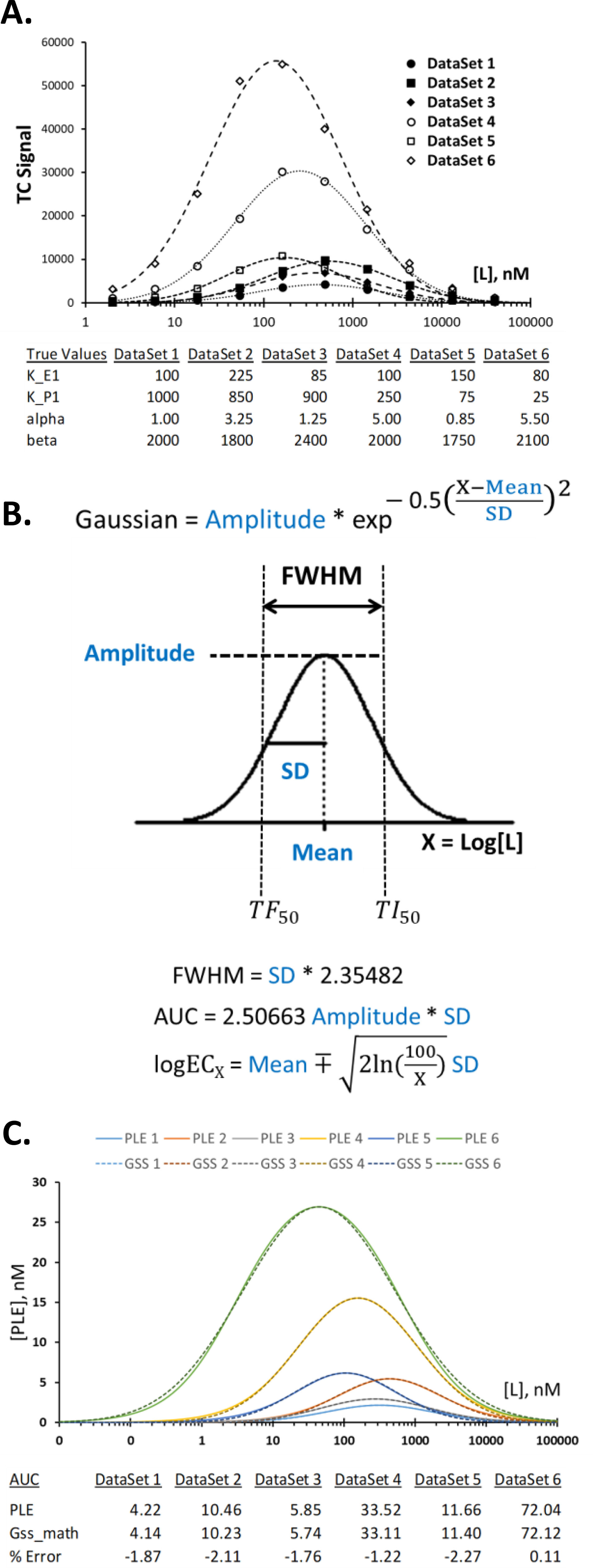
**Curve fitting of ternary complex dose-response data with a Gaussian function.** In the *top panel* of *A*, Gaussian function was used to fit the simulated dose-response data generated using the parameters shown in the *bottom panel* and mathematical equations in Fig. 1*B*. The *symbols* (*open* and *filled circles*, *squares*, and *diamonds*) are simulated data, and the *dotted lines* are predicted values from the Gaussian function curve fittings. Curve fitting was done by the iLSS method described in File S5 (*part 3*). The mathematical definition for the Gaussian function and some of the known mathematical properties of this function are shown in *B*. Although the mathematical solution for TF_50_ and TI_50_ in the PLE function is rather complex (File S4, *part 1*), use of the Gaussian function provides a very simple solution from the formula, TF_50_ (TI_50_) = 10^mean ∓ 1.17741SD (Equations 5 – 6*a* and 5 – 6*b* in File S5, part 1). More generally, the concentration that gives *X* percentage of the maximal response, defined as EC*_X_*, can be obtained by the formula shown in *B* (see File S5, *part 2*, for derivation), where *log* is log10, and *ln* is natural log. The validity of using Gaussian function to represent PLE curve is shown in *C*, where mathematically generated curve for PLE for each of the training data set is plotted in *solid lines* of different colors, and equivalent Gaussian curves are plotted in *dotted lines* of matching colors. AUC for the PLE function was obtained by dividing the entire curve into 700 segments of equal width and numerically adding areas of individual rectangles. The AUC for the Gaussian function (Gss_math) was obtained from the formula given in *B*.

The lack of mathematical description of the ternary complex system in the past led to a lack of proper analytical tools for quantitative analysis of experimental data. In practical terms, a curve-fitting method that can capture the geometric features of the dose-response curve as discussed in the previous section is highly desired. For this purpose, six different sets of simulated 12-point dose-response data were generated and used as a training data set. These data set have different *K*_P1_, *K*_E1_, and α values, covering a range of possibilities often found in the experimental settings (see the *bottom panel* of Fig. 5*A* for actual values). Because ternary complexes are often measured in arbitrary light units that provide no information on the actual concentration of the ternary complex, the simulated [PLE] data were converted to a unitless ternary complex signal (TCS) by introducing a system conversion factor, β, which also serves as a system calibration factor, according to the following relationship: TCS = β × [PLE].

The value of β depends on the specific measurement method and configuration of the ternary complex assay system. Once the system configuration is fixed, then the conversion factor β is expected to be largely unaffected by individual ligand, although small variations may occur because the tightness of the protein–protein interaction varies. The simulated training data set contains small variations in β to emulate this situation (Fig. 5*A*, *bottom panel*). In most experiments, the value of β is unknown unless the system is calibrated against an orthogonal method that provides absolute concentration of the ternary complex. Therefore, most experimentally obtained data will be TCS plotted against log of concentration of the TC-forming ligand.

Among different curve-fitting methods surveyed by the author, Gaussian function gave an excellent reproduction of the simulated data upon curve fitting (Fig. 5*A*). The Gaussian function on the semi-log scale consists of three independent parameters: amplitude, mean, and S.D. (Fig. 5*B*). Representation of the TCS data by the Gaussian curve allows utilization of established mathematical relationships within the Gaussian function for quantitative description of the ternary complex dose-response curve. For example, the AUC for the Gaussian curve is known to be AUC = 2.50663 amplitude × S.D. = 1.06447 amplitude × FWHM. The empirical formula found earlier (AUC ≅ 1.1 FWHM × [PLE]_max_) is consistent with this analytical formula of the Gaussian function.

To further test how well the Gaussian function can reproduce the ternary complex dose-response data, mathematical equivalence between the PLE function (Equations 2 – 1a and 2 – 1b in Fig. 1*B*) and the Gaussian function (Fig. 5*B*) was established (File S5, *part 1*). For each of the six simulated training data set in Fig. 5*A*, the expected [PLE] values were calculated from both functions (Fig. 5*C*). Despite a consistent but small deviation between the two curves, corresponding to a small difference in the slope of the curves, there was an excellent overall agreement between the two curves. The utility of the Gaussian function in representing the PLE function is shown by the close agreement of the two AUC values as shown in Fig. 5*C* (*bottom panel*). From these comparisons, it is concluded that, for all practical purposes, the Gaussian function can be used to fit the ternary complex dose-response data and quickly provide quantitative information on potency and efficacy of target engagement. Useful mathematical formula for this purpose are shown in Fig. 5*B* and File S5 (*part 1*).

### Obtaining equilibrium constants from the experimental ternary complex dose-response data: LeastSumSquare methods

Information on equilibrium constants such as *K*_P1_, *K*_E1_, and α usually requires separate measurements. Biophysical methods such as surface plasmon resonance or isothermal calorimetry are often used for this purpose (19). It would be extremely useful if these equilibrium constants can be extracted from appropriate curve fitting of the dose-response data without having to resort to external biophysical methods. The iterative LeastSumSquare method (iLSS) (File S6*A*) was evaluated for determining the equilibrium constants (*K*_P1_, *K*_E1_, and α) and the system conversion factor β that best described the experimental TCS data. The initial results from this iLSS method indicated the importance of knowing free ligand concentration at equilibrium for successful application of this method (data not shown). A numerical method was developed to calculate free ligand concentration at a given equilibrium concentration of the ternary complex (File S6*B*). The extended LeastSumSquare (extLSS) method (File S6*C*) incorporated numeric calculation of the free ligand concentrations in every iteration within the iLSS method. Using the same simulated training data set shown in Fig. 5*A*, 5% random errors were introduced in the TCS values and were used to evaluate the extLSS method. The results shown in File S6*E* demonstrated the proof of concept for this method. A template program for the extLSS method is provided as an Excel file in the supporting information (BHan_TCextLSS_v3.5.4_200221.xlsx).

## Conclusions

In summary, I provide a comprehensive suite of mathematical solutions for the ternary complex system and provide mechanistic explanations for many commonly encountered challenges ranging from theoretical understanding of the system to experimental measurements of key parameters. These tools can be used to answer many “what if” questions and will be helpful in troubleshooting challenges and making informed decisions for the direction of the SAR of small molecule protein degraders. A one-page summary of the major findings in this article is provided in File S7 for easy reference. Because these mathematical tools do not require any assumptions or restrictions on the system, the mathematical principles and tools introduced in this article will be applicable to wide range of ternary complex systems outside the targeted protein degradation field.

## Experimental procedures

### Kinetic simulation of the ternary complex formation

Kinetic simulation was done with a program written in Microsoft Excel, which is described in File S2. An example program is provided as a separate file in the supporting information (BHan_TCKinSim_v3.5.4_200505.xlsx). Before running the simulation, all of the equilibrium dissociation constants need to be reduced into individual forward and reverse rate constants, *k*_on_ and *k*_off_, respectively. The program takes the user-provided equilibrium constants (*K*_P1_, *K*_E1_, and α) and automatically assigns rate constants to each step in Fig. 2*A* following the simple set of rules described in File S2. The user can provide different rate constants or different rules as long as these values satisfy the mathematical relationships among each of the equilibrium constants (*K*_P1_, *K*_E1_, *K*_P2_, *K*_E2_, and α) as outlined in Fig. 1*B*. Using a different set of rate constants will change the kinetics of binding, but they do not affect the simulated equilibrium concentrations, which are dictated only by the equilibrium constants.

### Curve fitting of dose-response data by iLSS method and extLSS method

The iLSS method (described in File S5, *part 3*) was used for curve fitting of the simulated TCS dose-response data with Gaussian function using log10 values of total ligand concentrations. For curve fitting of the TCS dose-response data with the PLE function (Equations 2 – 1a and 2 – 1b in Fig. 1*B*), unmodified total ligand concentration was used as described in File S6*A*. File S6*B* describes a method to calculate free ligand concentration, and File S6*C* describes the extLSS method, which uses free ligand concentration for curve fitting of the dose-response data to extract equilibrium constants from these data. A template program for each of these methods is provided in an Excel file in the supporting information (BHan_TCextLSS_v3.5.4_200221.xlsx).

## Data availability

All the data used in this article are contained within the article and the supporting information.

## Supplementary Material

Supporting Information
