# Supplementary material for: A suite of mathematical solutions to describe ternary complex formation and their application to targeted protein degradation by heterobifunctional ligands
Source: J Biol Chem. 2020 Aug 28;295(45):15280–91. doi: 10.1074/jbc.RA120.014715 (PMC7650257; doi:10.1074/jbc.RA120.014715)
Supplement: Supporting Information [file supp_295_45_15280__index.html]

A suite of mathematical solutions to describe ternary complex formation and their application to targeted protein degradation by hetero-bifunctional ligands — Mathematical understanding of the ternary complex system — A suite of mathematical solutions to describe ternary complex formation and their application to targeted protein degradation by heterobifunctional ligands — Mathematical understanding of the ternary complex system — Supporting Information 

# A suite of mathematical solutions to describe ternary complex formation and their application to targeted protein degradation by heterobifunctional ligands

## Supporting Information

- Supporting Information (to be published online) - Complete mathematical models and equations
- Supporting Information (to be published online) - Revised version of the Excel-based template program for calculating equilibrium concentrations
- Supporting Information (to be published online) - Excel-based program for kinetic simulation of ternary complex binding
- Supporting Information (to be published online) - Excel-based template program for curve fitting of experimental dose-response data
