# Supplementary material for: A suite of mathematical solutions to describe ternary complex formation and their application to targeted protein degradation by heterobifunctional ligands
Source: J Biol Chem. 2020 Aug 28;295(45):15280–91. doi: 10.1074/jbc.RA120.014715 (PMC7650257; doi:10.1074/jbc.RA120.014715)

Supporting Information 1A. Mathematical Description of the Ternary Complex at Equilibrium

Key ternary complex equilibrium equations are given below followed by derivation of each of them.

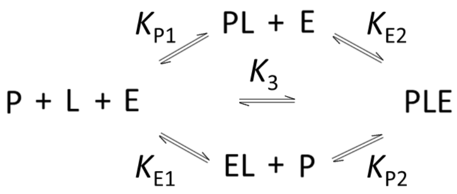

$$K_{P1} = \frac{[P] \cdot [L]}{[PL]} \qquad Eq. 1-1*$$

$$K_{E1} = \frac{[E] \cdot [L]}{[EL]} \qquad Eq. 1-2*$$

$$\alpha \equiv \frac{K_{P1}}{K_{P2}} = \frac{K_{E1}}{K_{E2}} \qquad Eq. 1-3*$$

α, cooperativity factor
> 1 for positive cooperation

$$K_{P2} = \frac{[P] \cdot [EL]}{[PLE]} \qquad Eq. 1-4$$

$$K_{E2} = \frac{[PL] \cdot [E]}{[PLE]} \qquad Eq. 1-5$$

$$K_3 = \frac{[P] \cdot [L] \cdot [E]}{[PLE]} \qquad Eq. 1-6$$

$$[P] = ([P_t] - [PLE]) \frac{K_{P1}}{[L] + K_{P1}} \qquad Eq. 1-7$$

$$[PL] = ([P_t] - [PLE]) \frac{[L]}{[L] + K_{P1}} \qquad Eq. 1-8$$

$$[E] = ([E_t] - [PLE]) \frac{K_{E1}}{[L] + K_{E1}} \qquad Eq. 1-9$$

$$[EL] = ([E_t] - [PLE]) \frac{[L]}{[L] + K_{E1}} \qquad Eq. 1-10$$

$$[PLE] = \frac{\left[f([L]) - \sqrt{f^2([L]) - 4[P_t] \cdot [E_t]}\,\right]}{2}$$
$$Eq. 2-1a$$

$$f([L]) = [P_t] + [E_t] + \frac{1}{\alpha \cdot [L]}([L] + K_{P1})([L] + K_{E1})$$
$$Eq. 2-1b$$

An equilibrium binding of a hetero-bifunctional ligand (L) with its target protein (P) and an E3 ligase (E) can be described as a series of bimolecular binding reactions as depicted above. The ligand, L, can initially form a binary complex either with the target protein, P, or with an E3 ligase, E, to form PL or EL with an equilibrium dissociation constant of K_{P1} and K_{E1}, respectively (Eq.1-1 and Eq.1-2). Each of these bimolecular complexes in turn can bind with E or P to form a ternary complex PLE with an equilibrium dissociation constant of K_{E2} and K_{P2}, respectively (Eq.1-5 and Eq.1-4). Alternatively, all three of the L, P, and E may simultaneously come together to form a ternary complex in one step with an equilibrium dissociation constant of K_3 (Eq.1-6). While there are up to five different binding reactions in this system, the equilibrium dissociation constants are constrained in certain ways dictated by the thermodynamic principle of pathway independence. In other words, the overall equilibrium constant, K_{PLE}, is dictated only by the difference in the free energy status between the final and initial states and is independent of the specific path. The following equation captures this principle.

$$K_{PLE} = K_{P1} \cdot K_{E2} = K_{E1} \cdot K_{P2} = K_3$$

Indeed, substituting equations 1-1,1-2,1-4, and 1-5 into the above expression confirms that the overall equilibrium dissociation constant K_{PLE} turns out to be the same regardless of the specific pathway.

$$K_{PLE} = \frac{[P]\cdot[L]}{[PL]}\cdot\frac{[PL]\cdot[E]}{[PLE]} = \frac{[E]\cdot[L]}{[EL]}\cdot\frac{[P]\cdot[EL]}{[PLE]} = \frac{[P]\cdot[L]\cdot[E]}{[PLE]}$$

With these constraints on the equilibrium dissociation constants, the ternary complex system can be completely described by three independent equilibrium constants, K_{P1}, K_{E1}, and α, as described by equations, Eq.1-1, E1.1-2, and Eq.1-3.

First, we find a few simple equilibrium relationships involving the target protein, P, below.

At all times, total concentration of all forms of the target protein species, P_t, should stay the same.

$$P_t = [P] + [PL] + [PLE] \qquad \text{Supp. Eq. 1-1}$$

Substituting [PL] in the Supp. Eq. 1-1 with Eq. 1-1,

$$K_{P1} = \frac{[P]\cdot[L]}{[PL]} \qquad Eq. 1-1$$

$$[P_t] = [P] + [P]\frac{[L]}{K_{P1}} + [PLE] = [P]\frac{K_{P1}+[L]}{K_{P1}} + [PLE]$$

$$[P_t] - [PLE] = [P]\frac{[L]+K_{P1}}{K_{P1}}$$

Rearranging the equation above, we obtain

$$[P] = ([P_t] - [PLE]) \frac{K_{P1}}{[L] + K_{P1}} \qquad Eq.1-7$$

Similarly, by substituting [P] in the Supp. Eq. 1-1 with Eq. 1-1, and solving for [PL], we obtain

$$[PL] = ([P_t] - [PLE]) \frac{[L]}{[L] + K_{P1}} \qquad Eq.1-8$$

Next, we find a few similar simple equilibrium relationships involving the E3 ligase, E, following the same steps as described above for the target protein, P. E_t is the total concentration of all forms of the E3 ligase species.

$$[E] = ([E_t] - [PLE]) \frac{K_{E1}}{[L] + K_{E1}} \qquad Eq.1-9$$

$$[EL] = ([E_t] - [PLE]) \frac{[L]}{[L] + K_{E1}} \qquad Eq.1-10$$

Now, we can turn to Eq. 1-6 for equilibrium expression of the [PLE], and substitute K_3 with $K_{P1} \cdot K_{E2}$ per thermodynamic principle of pathway independence of the equilibrium constant to obtain the followings.

$$[PLE] = \frac{[P] \cdot [L] \cdot [E]}{K_3} \qquad from\ Eq.1-6$$

$$= \frac{[P] \cdot [L] \cdot [E]}{K_{P1} \cdot K_{E2}} \qquad from\ thermodynamic\ principle\ of\ pathway\ independenc$$

Substituting K_{E2} with K_{E1}/α into above equation, we obtain Supp.Eq.1-3 below

$$[PLE] = \frac{[P] \cdot [L] \cdot [E] \cdot \alpha}{K_{P1} \cdot K_{E1}} = \alpha \cdot [L] \frac{[P]}{K_{P1}} \cdot \frac{[E]}{K_{E1}} \qquad Supp.Eq.1-3$$

[P] and [E] in the above equation can be substituted by Eq. 1-7 and Eq. 1-9, respectively, to obtain

$$[PLE] = \alpha \cdot [L] \frac{[P_t] - [PLE]}{[L] + K_{P1}} \cdot \frac{[E_t] - [PLE]}{[L] + K_{E1}} \qquad Supp.Eq.1-4$$

The above equation can be rearranged as below in the form of the second order polynomial for [PLE].

$$([P_t] - [PLE])([E_t] - [PLE]) = \frac{1}{\alpha \cdot [L]} [PLE]\,([L] + K_{P1})([L] + K_{E1})$$

$$[PLE]^2 - \{[P_t] + [E_t] + \frac{1}{\alpha \cdot [L]}([L] + K_{P1})([L] + K_{E1})\}\,[PLE] + [P_t] \cdot [E_t] = 0$$

Notice that P_t, E_t, K_{P1}, K_{E1}, and α are all constants unique to the system. Therefore, at any given value of [L], the only variable in the above equation is [PLE]. Since the equation is a quadratic function for [PLE], exact solution for [PLE] can be obtained using quadratic formula as

$$[PLE] = \left[[P_t] + [E_t] + \frac{1}{\alpha \cdot [L]}([L] + K_{P1})([L] + K_{E1}) \pm \sqrt{\{[P_t] + [E_t] + \frac{1}{\alpha \cdot [L]}([L] + K_{P1})([L] + K_{E1})\}^{\wedge}2 - 4\,[P_t] \cdot [E_t]}\right]/2$$

Since [PLE] cannot be bigger than ($[P_t]$ + $[E_t]$)/2, the only valid solution for [PLE] is

$$[PLE] = \left[[P_t] + [E_t] + \frac{1}{\alpha \cdot [L]}([L] + K_{P1})([L] + K_{E1}) - \sqrt{\{[P_t] + [E_t] + \frac{1}{\alpha \cdot [L]}([L] + K_{P1})([L] + K_{E1})\}^{\wedge}2 - 4\,[P_t] \cdot [E_t]}\right]/2$$

Alternatively, [PLE] can be written as

$$[PLE] = \left[f([L]) - \sqrt{f^2([L]) - 4\,[P_t] \cdot [E_t]}\right]/2 \qquad Eq.\ 2-1a$$

$$where\ f([L]) = [P_t] + [E_t] + \frac{1}{\alpha \cdot [L]}([L] + K_{P1})([L] + K_{E1}) \qquad Eq.\ 2-1b$$

Supporting Information 1B. Iterative method 1 to calculate equilibrium concentrations of each species as a function of the total, as opposed to free, ligand concentration

If we use the total ligand concentration, $[L]_{total}$, to calculate concentrations of PL, EL and PLE according to the equations Eq.1-8, 1-10, and 2-1a/b above, respectively, then calculated total ligand concentration, $[L]_{total\_calculated}$, will exceed the true total ligand concentration, $[L]_{total\_true}$.

$$[L]_{total\_calculated} = [L]_{total} + [PL] + [EL] + [PLE] > [L]_{total\_true}$$

Free ligand concentration at equilibrium, $[L]_{free}$, is lower than total ligand concentration due to consumption of some by participating in formation of PL, EL, and PLE. If we pick a number, $[L]_1$, that is lower than $[L]_{total\_true}$ but higher than $[L]_{free}$, and recalculate concentrations of each species using the same equations, then the recalculated total ligand concentration, $[L]_{total\_1}$, will be still higher than the true $[L]_{total}$.

$$[L]_{total\_1} = [L]_1 + [PL]_1 + [EL]_1 + [PLE]_1 > [L]_{total\_true}$$

If we pick another number, $[L]_2$, that is lower than $[L]_{free}$, then the recalculated total ligand concentration, $[L]_{total\_2}$, will be lower than the true $[L]_{total}$.

$$[L]_{total\_2} = [L]_2 + [PL]_2 + [EL]_2 + [PLE]_2 < [L]_{total\_true}$$

In the third round, we can make an educated guess for the $[L]_3$ based on the results from the previous two rounds and pick a number between $[L]_1$ and $[L]_2$. This process of picking an estimated value for $[L]_{free}$ and modifying the trial value of $[L]_{free}$ can keep going on until the calculated $[L]_{total}$ becomes equal to, or sufficiently close to, true $[L]_{total}$. This iterative process can be automatically repeated until a preset conversion criterion is met using Solver function within Microsoft Excel. A template method is provided in a separate file, **BHan_PLEcalc_v1.2_200727.xlsx**. Detailed instruction on how to use this template is provided within the Excel file. Schematic algorithm for this method is shown below.

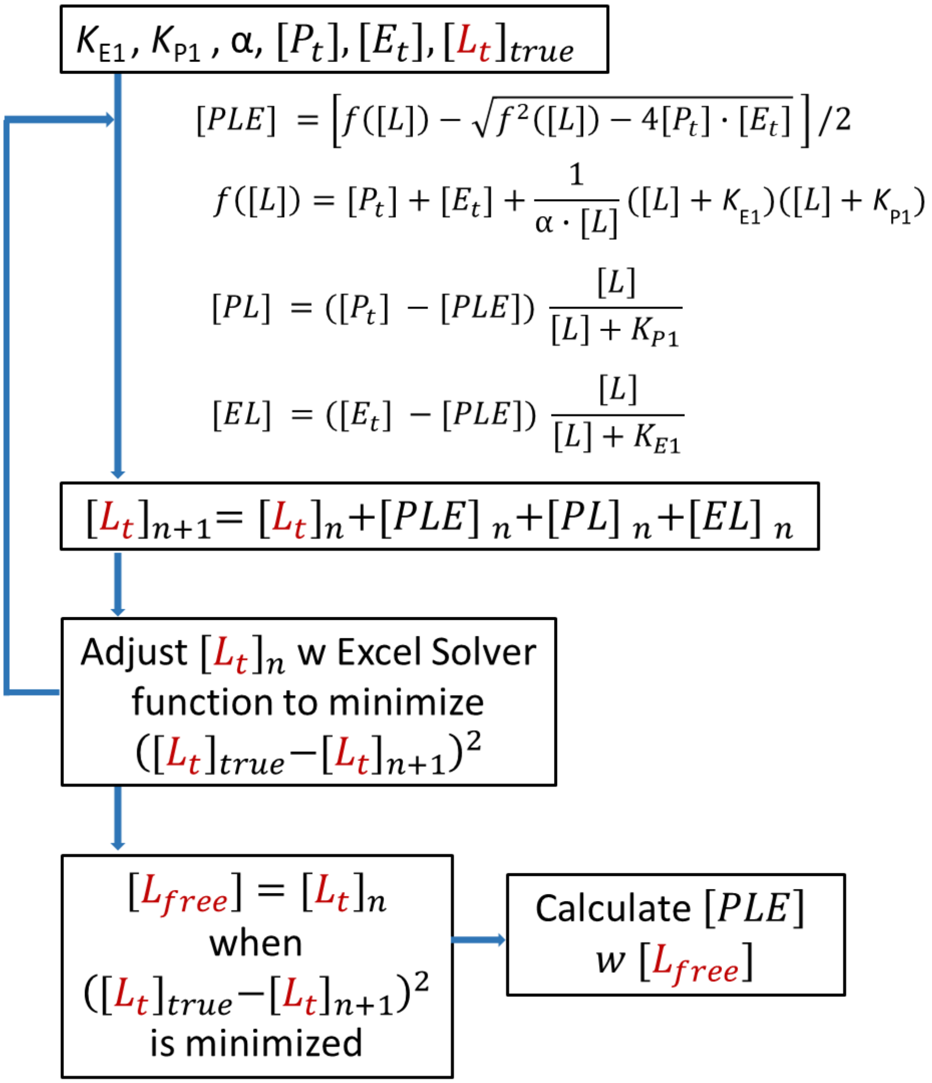
K_{E1}, K_{P1}, α, $[P_t]$, $[E_t]$, $[L_t]_{true}$
$[PLE] = \left[f([L]) - \sqrt{f^2([L]) - 4[P_t]\cdot[E_t]}\right]/2$
$f([L]) = [P_t] + [E_t] + \frac{1}{\alpha \cdot [L]}([L] + K_{E1})([L] + K_{P1})$
$[PL] = ([P_t] - [PLE])\frac{[L]}{[L] + K_{P1}}$
$[EL] = ([E_t] - [PLE])\frac{[L]}{[L] + K_{E1}}$
$[L_t]_{n+1} = [L_t]_n + [PLE]_n + [PL]_n + [EL]_n$
Adjust $[L_t]_n$ w Excel Solver function to minimize $([L_t]_{true} - [L_t]_{n+1})^2$
$[L_{free}] = [L_t]_n$ when $([L_t]_{true} - [L_t]_{n+1})^2$ is minimized
Calculate $[PLE]$ w $[L_{free}]$

Supporting Information 2. Excel-based kinetic simulation program for ternary complex formation.

An Excel-based program for kinetic simulation can be found in

BHan_TCKinSim_v3.5.4_200505.xlsx

Instruction on how to use the program is provided in the "Instruction" tab.

There are twelve hidden tabs, labeled as "**DR1**" through "**DR12**". Do not modify them. The program is run within the "**Run**" tab, which serves as a control panel. All user-provided data and parameters are entered in this tab only within the areas highlighted with bright yellow.

A backup copy of the control panel is in the "**Run_backup**" tab. If restoration of default settings is desired or if the program codes get corrupted within the control panel, them make a copy of the desired area from the backup tab and paste into the "**Run**" tab.

Typical simulation exercise can be done in four steps.

1. Initiation of the simulation w precision value of 0.1, or 10% maximum error.
2. Iterative simulation w precision value of 0.01 in the circular reference mode.
3. 1st refinement w precision 0.001
4. Optional 2nd refinement w precision 0.0001

Contact Bomie Han at bomiehan@lilly.com for further questions/instructions. Additional details on how the program works are provided below.

This program contains 12 copies of the identical module that allows simultaneous execution of 12 different reaction conditions, which can be used to simulate a 12-point dose-response experiment. These 12 modules are controlled from a central panel within the tab, labeled as "Run". Each module contains a block of 1000 simulation steps, which can be looped into a circular reference so that one cycle of simulation can go through hundreds of blocks of simulation steps without interference. A typical simulation experiment consists of running one block of simulation followed by three cycles of simulation with increasing precision and decreasing speed at each cycle. Before running the simulation cycles, basic information on the experimental condition is provided within the central panel such as the equilibrium constants (K_{P1}, K_{E1}, and α) and concentrations of individual protein and the ligand. From the three independent equilibrium constants, all the other equilibrium dissociation constants in the **Figure 2*A*** are calculated. Each of these equilibrium dissociation constants (K_d) need to be broken down into an association and a dissociation rate constant (k_on and k_off, respectively) in a way to satisfy the relationship of K_d = k_off/k_on. The program provides suggested values based on a simple set of rules

show below to maintain consistency across different parts of the system: 1) The ratio of the forward (k_on) and reverse (k_off) rate constants and the equilibrium dissociation constant (Kd) for any given binding reaction must satisfy the equation, K_d = k_off / k_on. 2) The value for the rate constant k_off follows the rule k_off = ln2/$t_{1/2}$, and the half time of dissociation ($t_{1/2}$) is 15 sec for equilibrium dissociation constant of 100 nM. 3) Change in K_d values from 100 nM is reflected equally between decrease in k_on and increase in k_off. As a result, half time of dissociation for X nM K_d is SquareRoot(X nM/100 nM) * 15 sec. The user can enter any value as long as the thermodynamic equilibrium principle is not broken.

Once the kinetic simulation is initiated, a small test value of time interval, delta t, is selected and changes in concentration of each component is calculated for the time interval. These values are compared to the pre-set user definable parameters that dictate the speed and precision of simulation. If overall changes in concentration are bigger than maximum value allowed by the simulation precision, then the test time interval is scaled back. If the overall changes in concentration are smaller than this preset value, then a bigger time interval is taken within limits dictated by the maximum simulation speed. The re-calculated changes in concentration of each component within the adjusted time interval is fed into the concentration of each component and the elapsed time as well as the optimized time interval for the simulation cycle is recorded. This completes one step in the simulation process. In the next step, simulation starts with the previously optimized time interval and repeats the whole process. As the reaction progresses and concentrations of individual components change, the optimized time interval keeps changing for each cycle but always starts with the optimized time interval for the previous step. In this manner, the simulation proceeds with the maximum efficiency allowed within the preset precision throughout the whole course of the simulation. Since each simulation cycle records total time elapsed, simulations can be tracked by the reaction time as well as by the number of simulation steps.

For most efficient execution of simulation with the maximum precision, the whole simulation exercise is broken into four different cycles; initiation, execution, refinement, and optional fine refinement. The first cycle of “initiation” consists of one block of 1000 steps of simulation with a precision factor of 0.1, corresponding to maximum allowed change in concentration at any simulation step is 10%. At the end of the initiation cycle, the circular reference is engaged by feeding the outcome of the block of 1000 steps as the initial condition into next block of simulation steps. The second cycle of “execution” consists of repetition of up to 500 blocks (500,000 simulation steps) with a precision factor of 0.01, or 1% maximal change in concentration per simulation step. The third cycle of “refinement” is done in the same manner with a precision factor of 0.001. The optional fourth cycle of “fine refinement” is done with a precision factor of 0.0001. Detailed instruction is provided in the “Instruction” tab within the Excel-based program file. Outcomes of each simulation cycle can be followed from graphical output of the results built in the program.

Supporting Information 3-1, Common variations of the ternary complex system with additional equilibria

Three common variations of the ternary complex system are shown below along with mathematical methods to handle them. In ***A***, the target protein P is in conformational equilibrium with a closed form, P_c, with an equilibrium constant K_c as defined in Eq. 3-1a. P_c cannot bind the ligand. In ***B***, there is an endogenous ligand, C, that binds to the target protein with an equilibrium dissociation constant K_i as described in Eq. 3-2a. The endogenous ligand C competes with the exogenous hetero-bifunctional ligand L. In ***C***, the two extra equilibria described above coexist. In each case, the equilibrium concentration of the ternary complex can be calculated by the equations Eq. 3-4a and 3-4b in ***D***. Notice that these equations are identical to the equations Eq. 2-1a & 2-1b in **Figure 1B** for the simple ternary complex system except that the binary dissociation constant between the hetero-bifunctional ligand and the target protein, K_{p1}, was replaced with K'_{p1} as shown in equations Eq. 3-1b, Eq. 3-2b, and Eq. 3-3c, respectively. Full mathematical derivation for each of these equations are shown in **Supporting Information 3-2A**, **3-2B**, and **3-2C**, respectively. Mathematical equations for similar cases in the binary complex system are shown in **Supporting Information 3-3** as a comparison.

A.

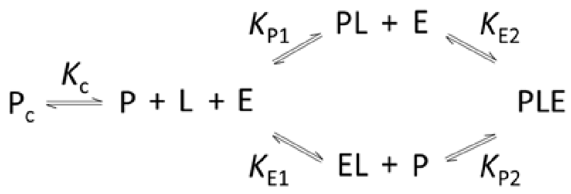

$$K_c = \frac{[P_C]}{[P]} \qquad Eq.3-1a$$

$$K'_{P1} = K_{P1}(1 + K_c) \qquad Eq.3-1b$$

$$= \frac{([P] + [P_c]) \cdot [L]}{[PL]} \qquad Eq.3-1c$$

B.

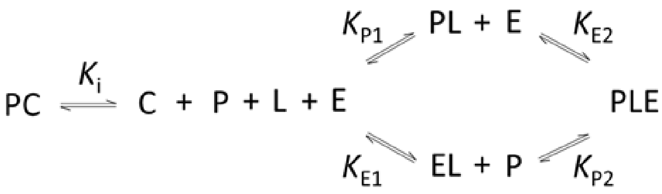

$$K_i = \frac{[P] \cdot [C]}{[PC]} \qquad Eq.3-2a$$

$$K'_{P1} = K_{P1}\left(1 + \frac{[C]}{K_i}\right) \qquad Eq.3-2b$$

$$= \frac{([P] + [PC]) \cdot [L]}{[PL]} \qquad Eq.3-2c$$

C.

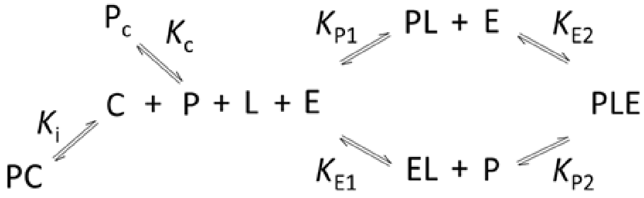

$$K_c = \frac{[P_C]}{P} \qquad Eq.3-3a$$

$$K_i = \frac{[P] \cdot [C]}{[PC]} \qquad Eq.3-3b$$

$$K'_{P1} = K_{P1}\left(1 + K_c + \frac{[C]}{K_i}\right) \qquad Eq.3-3c$$

$$= \frac{([P] + [P_c] + [PC]) \cdot [L]}{[PL]} \qquad Eq.3-3d$$

D.

$$[PLE] = \left[f([L]) - \sqrt{f^2([L]) - 4[P_t] \cdot [E_t]}\,\right]/2 \qquad Eq.3-4a$$

$$f([L]) = [P_t] + [E_t] + \frac{1}{\alpha \cdot [L]}([L] + K'_{P1})([L] + K_{E1}) \qquad Eq.3-4b$$

Supporting Information 3-2A. Case for a conformational equilibrium of the target protein

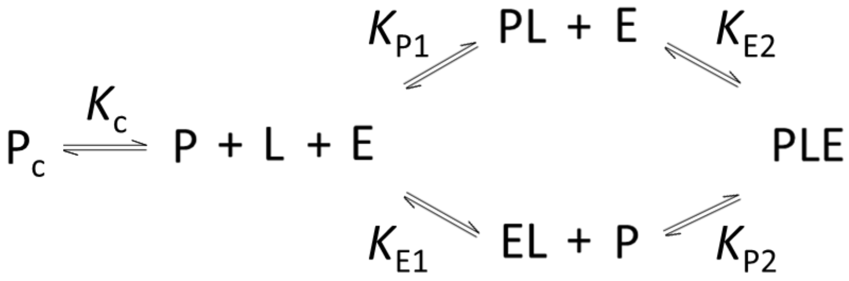

When there is an additional equilibrium involving the target protein between closed conformation, P_c, and the open conformation, P, with an equilibrium constant of K_c,

$$K_c = \frac{[P_c]}{[P]} \qquad Eq. 3-1a$$

of which only the open conformation can bind the ligand, total concentration of the target protein, P_t, can be written as

$$[P_t] = [P_c] + [P] + [PL] + [PLE] \qquad Supp. Eq. 3-1a$$

$$= [P](1 + K_c) + [PL] + [PLE] \qquad from\ Eq. 3-1a$$

$$= [P](1 + K_c) + [P]\frac{[L]}{K_{P1}} + [PLE] \qquad from\ Eq. 1-1$$

By defining K'_{P1} as below in Eq.3-1b,

$$K'_{P1} = K_{P1}(1 + K_c) \qquad Eq. 3-1b$$

$[P_t]$ can be written as below,

$$[P_t] = [P]\frac{K'_{P1} + [L]}{K_{P1}} + [PLE]$$

leading to

$$[P] = ([P_t] - [PLE])\frac{K_{P1}}{[L] + K'_{P1}}$$

Rearranging the above equation, we obtain,

$$\frac{[P]}{K_{P1}} = \frac{[P_t] - [PLE]}{[L] + K'_{P1}} \qquad Supp.Eq.3-1b$$

By substituting Supp.Eq.3-1b into Supp. Eq.1-3,

$$[PLE] = \frac{[P]\cdot[L]\cdot[E]\cdot\alpha}{K_{P1}\cdot K_{E1}} = \alpha\cdot[L]\,\frac{[P]}{K_{P1}}\cdot\frac{[E]}{K_{E1}} \qquad Supp.Eq.1-3$$

[PLE] can be written as

$$[PLE] = \frac{[P]\cdot[L]\cdot[E]\cdot\alpha}{K_{P1}\cdot K_{E1}} = \alpha\cdot[L]\,\frac{[P_t] - [PLE]}{[L] + K'_{P1}}\cdot\frac{[E]}{K_{E1}} \qquad Supp.Eq.3-1c$$

$[E]/K_{E1}$ above can be substituted by Eq.1-9 to yield,

$$[PLE] = \frac{[P]\cdot[L]\cdot[E]\cdot\alpha}{K_{P1}\cdot K_{E1}} = \alpha\cdot[L]\,\frac{[P_t] - [PLE]}{[L] + K'_{P1}}\cdot\frac{[E_t] - [PLE]}{[L] + K_{E1}} \qquad Supp.Eq.3-1d$$

Notice that Supp.Eq.3-1d is identical to Supp.Eq.1-4 except that K_{P1} is substituted with K'_{P1}. The same steps can be followed as in the case for Supporting Information 1 to obtain,

$$[PLE] = \left[f([L]) - \sqrt{f^2([L]) - 4\,[P_t]\cdot[E_t]}\,\right]/2 \qquad Eq.\ 3-4a$$

$$where\ \ f([L]) = [P_t] + [E_t] + \frac{1}{\alpha\cdot[L]}([L] + K'_{P1})([L] + K_{E1}) \qquad Eq.\ 3-4b$$

Supporting Information 3-2B. Case for a competition by a mono-functional ligand for the target protein

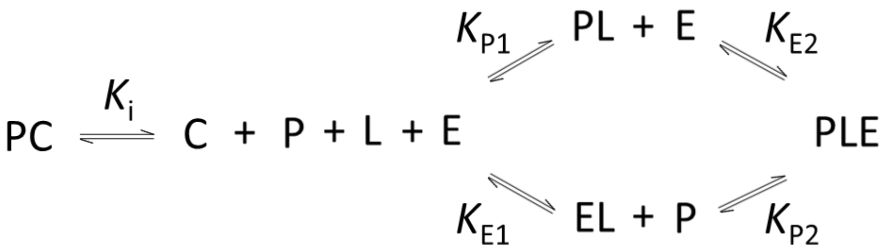

When there is an additional equilibrium involving the target protein binding a mono-functional ligand, or a competitor, C, with an equilibrium constant of K_i,

$$K_i = \frac{[P] \cdot [C]}{[PC]} \qquad Eq. 3-2a$$

total concentration of the target protein, P_t, can be written as

$$[P_t] = [PC] + [P] + [PL] + [PLE] \qquad Supp. Eq. 3-2a$$

$$[P_t] = [P]\left(1 + \frac{[C]}{K_i}\right) + [PL] + [PLE] \qquad from\, Eq. 3-2a$$

$$= [P]\left(1 + \frac{[C]}{K_i}\right) + [P]\frac{[L]}{K_{P1}} + [PLE] \qquad from\, Eq. 1-1$$

By defining K'_{P1} as below in Eq.3-2b,

$$K'_{P1} = K_{P1}\left(1 + \frac{[C]}{K_i}\right) \qquad Eq. 3-2b$$

$[P_t]$ can be written as below,

$$[P_t] = [P]\frac{K'_{P1} + [L]}{K_{P1}} + [PLE]$$

leading to

$$[P] = ([P_t] - [PLE])\frac{K_{P1}}{[L] + K'_{P1}}$$

Rearranging the above equation, we obtain,

$$\frac{[P]}{K_{P1}} = \frac{[P_t] - [PLE]}{[L] + K'_{P1}} \qquad Supp.Eq.3-2b$$

By substituting Supp.Eq.3-2b into Supp. Eq.1-3,

$$[PLE] = \frac{[P] \cdot [L] \cdot [E] \cdot \alpha}{K_{P1} \cdot K_{E1}} = \alpha \cdot [L] \frac{[P]}{K_{P1}} \cdot \frac{[E]}{K_{E1}} \qquad Supp.Eq.1-3$$

[PLE] can be written as

$$[PLE] = \frac{[P] \cdot [L] \cdot [E] \cdot \alpha}{K_{P1} \cdot K_{E1}} = \alpha \cdot [L] \frac{[P_t] - [PLE]}{[L] + K'_{P1}} \cdot \frac{[E]}{K_{E1}} \qquad Supp.Eq.3-2c$$

$[E]/K_{E1}$ above can be substituted by Eq.1-9 to yield,

$$[PLE] = \frac{[P] \cdot [L] \cdot [E] \cdot \alpha}{K_{P1} \cdot K_{E1}} = \alpha \cdot [L] \frac{[P_t] - [PLE]}{[L] + K'_{P1}} \cdot \frac{[E_t] - [PLE]}{[L] + K_{E1}} \qquad Supp.Eq.3-2d$$

Notice that Supp.Eq.3-2d is identical to Supp.Eq.1-4 except that K_{P1} is substituted with K'_{P1}. The same steps can be followed as in the case for Supporting Information 1 to obtain,

$$[PLE] = \left[f([L]) - \sqrt{f^2([L]) - 4\,[P_t] \cdot [E_t]}\,\right]/2 \qquad Eq.\ 3-4a$$

$$where\ \ f([L]) = [P_t] + [E_t] + \frac{1}{\alpha \cdot [L]}([L] + K'_{P1})([L] + K_{E1}) \qquad Eq.\ 3-4b$$

Supporting Information 3-2C. Case for simultaneous extra equilibria involving conformational equilibrium of the target protein and competition by a mono-functional ligand

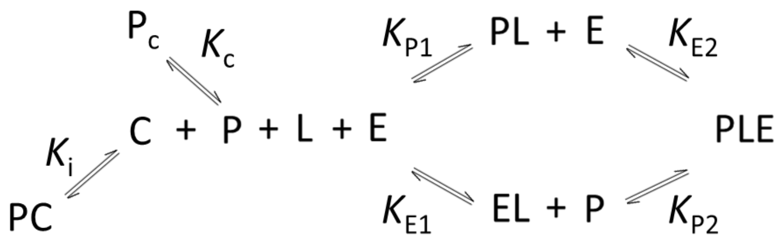

Certain kinases, for example, have both types of extra equilibria involving conformational equilibrium of the target protein and competition by endogenous ligand. With each of these extra equilibria described by Eq.3-3a and Eq.3-3b,

$$K_c = \frac{[P_c]}{[P]} \qquad Eq.3-3a$$

$$K_i = \frac{[P]\cdot[C]}{[PC]} \qquad Eq.3-3b$$

total concentration of the target protein, P_t, can be written as

$$[P_t] \ = [P_c] + [PC] + \ [P] + [PL] + [PLE] \qquad Supp.Eq.3-3a$$

$$[P_t] = [P]\cdot\ K_c + [P]\cdot\frac{[C]}{K_i} + [P] + [PL] + [PLE] \qquad from\ Eq.3-3a\ \&-3b$$

$$= \ [P]\cdot\ K_c + [P]\cdot\frac{[C]}{K_i} + [P] + [P]\frac{[L]}{K_{P1}} + [PLE] \qquad from\ Eq.1-1$$

By defining K'_{P1} as below in Eq.3-3c,

$$K'_{P1} = K_{P1}\left(1 + K_c + \frac{[C]}{K_i}\right) \qquad Eq.3-3c$$

$[P_t]$ can be written as below,

$$[P_t] \ = [P]\frac{K'_{P1} + [L]}{K_{P1}} + [PLE]$$

leading to

$$[P] = ([P_t] - [PLE]) \frac{K_{P1}}{[L] + K'_{P1}}$$

Rearranging the above equation, we obtain,

$$\frac{[P]}{K_{P1}} = \frac{[P_t] - [PLE]}{[L] + K'_{P1}} \qquad Supp.Eq.3-3b$$

By substituting Supp.Eq.3-3b into Supp. Eq.1-3,

$$[PLE] = \frac{[P] \cdot [L] \cdot [E] \cdot \alpha}{K_{P1} \cdot K_{E1}} = \alpha \cdot [L] \frac{[P]}{K_{P1}} \cdot \frac{[E]}{K_{E1}} \qquad Supp.Eq.1-3$$

[PLE] can be written as

$$[PLE] = \frac{[P] \cdot [L] \cdot [E] \cdot \alpha}{K_{P1} \cdot K_{E1}} = \alpha \cdot [L] \frac{[P_t] - [PLE]}{[L] + K'_{P1}} \cdot \frac{[E]}{K_{E1}} \qquad Supp.Eq.3-3c$$

$[E]/K_{E1}$ above can be substituted by Eq.1-9 to yield,

$$[PLE] = \frac{[P] \cdot [L] \cdot [E] \cdot \alpha}{K_{P1} \cdot K_{E1}} = \alpha \cdot [L] \frac{[P_t] - [PLE]}{[L] + K'_{P1}} \cdot \frac{[E_t] - [PLE]}{[L] + K_{E1}} \qquad Supp.Eq.3-3d$$

Notice that Supp.Eq.3-3d is identical to Supp.Eq.1-4 except that K_{P1} is substituted with K'_{P1}. The same steps can be followed as in the case for Supporting Information 1 to obtain,

$$[PLE] = \left[f([L]) - \sqrt{f^2([L]) - 4\,[P_t] \cdot [E_t]} \right] /2 \qquad Eq.\ 3-4a$$

$$where\ \ f([L]) = [P_t] + [E_t] + \frac{1}{\alpha \cdot [L]}([L] + K'_{P1})([L] + K_{E1}) \qquad Eq.\ 3-4b$$

Supporting Information 3-3. Mathematical equations for Binary Complex System with Additional Equilibrium as a comparison

$$P + L \underset{}{\overset{K_d}{\rightleftharpoons}} PL$$

$$K_d = \frac{[P]\,[L]}{[PL]} = \frac{k_{off}}{k_{on}}$$

$$[P_t] = [P] + [PL] = [PL]\,K_d\,/\,[L] + [PL]$$

$$[PL]\,(1 + K_d\,/\,[L]\,) = [P_t]$$

$$[PL] = [P_t]\,\frac{[L]}{[L] + K_d}$$

Conformational equilibrium

$$P_c + L \overset{K_{conf}}{\rightleftharpoons} P + L \overset{K_d}{\rightleftharpoons} PL$$

$$K_{conf} = \frac{[P_c]}{[P]}$$

$$K_{d,\,obs} = K_d\,(1 + K_{conf})$$

$$[P_t] = [P] + [P_c] + [PL] = [P](1+K_{conf}) + [PL]$$

$$= [PL](1 + K_{conf})\,K_d\,/\,[L]) + [PL]$$

$$[PL] = [P_t]\,\frac{[L]}{[L] + K_d\,(1+K_{conf})} = [P_t]\,\frac{[L]}{[L] + K_{d,obs}}$$

Competition

$$PC \overset{K_i}{\rightleftharpoons} P + L + C \overset{K_d}{\rightleftharpoons} PL$$

$$K_i = \frac{[P]\,[C]}{[PC]}$$

$$K_{d,\,obs} = K_d\,(1 + [C]/K_i)$$

$$[P_t] = [P] + [PC] + [PL] = [P](1+[C]/K_i) + [PL]$$

$$= [PL](1+[C]/K_i)\,K_d\,/\,[L] + [PL]$$

$$[PL] = [P_t]\,\frac{[L]}{[L] + K_d\,(1+[C]/K_i)} = [P_t]\,\frac{[L]}{[L] + K_{d,obs}}$$

Supporting Information 4-1. Geometric properties of the ternary complex dose-response curve

Summary of geometric properties of the typical ternary complex dose-response curve are shown below. Derivation for the mathematical equations for EC_{max}, $[PLE]_{max}$, and EC_{50} is shown in Supporting Information 4-2A, 4-2B, and 4-2C, respectively. Eq.4-5 for AUC (Area Under the Curve) is an empirical formula obtained from multiple simulated cases.

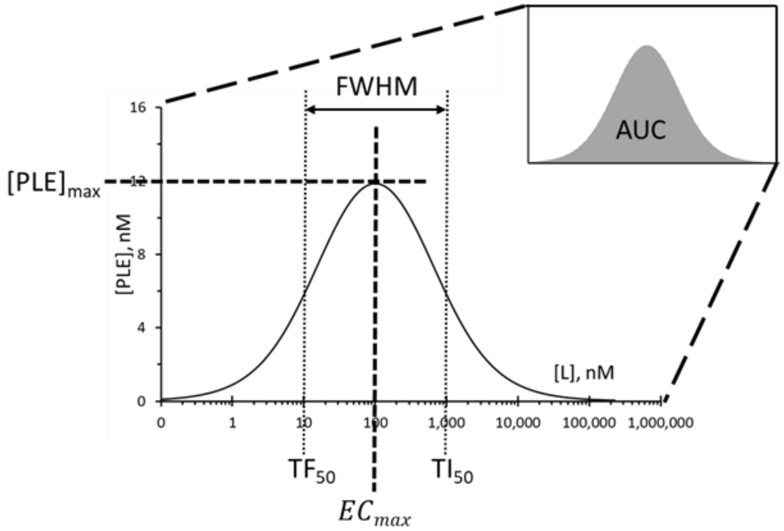

$$EC_{max} = \sqrt{K_{P1} \cdot K_{E1}} \qquad Eq. 4-1a$$

$$K_{P1} = {EC_{max}}^2 / K_{E1} \qquad Eq. 4-1b$$

$$\log(EC_{max}) = (\log K_{P1} + \log K_{E1}) / 2 \qquad Eq. 4-1c$$

$$[PLE]_{max} = \frac{\left[f(EC_{max}) - \sqrt{f^2(EC_{max}) - 4\,[P_t] \cdot [E_t]} \,\right]}{2} \qquad Eq. 4-2a$$

$$f(EC_{max}) = [P_t] + [E_t] + \left(\sqrt{K_{P1}} + \sqrt{K_{E1}}\right)^2/\alpha \qquad Eq. 4-2b$$

$$= [P_t] + [E_t] + (K_{P1} + K_{E1} + 2EC_{max})/\alpha \qquad Eq. 4-2c$$

$$\mathrm{EC}_{50} = \left[g \mp \sqrt{g^2 - 4\, K_{\mathrm{P1}} \cdot K_{\mathrm{E1}}} \,\right]/2 \qquad Eq. 4-3$$

$$\mathrm{TF}_{50} = \left[g - \sqrt{g^2 - 4\, K_{\mathrm{P1}} \cdot K_{\mathrm{E1}}} \,\right]/2 \qquad Eq. 4-3a$$

$$\mathrm{TI}_{50} = \left[g + \sqrt{g^2 - 4\, K_{\mathrm{P1}} \cdot K_{\mathrm{E1}}} \,\right]/2 \qquad Eq. 4-3b$$

$$g = \left(\frac{PLE_{\mathrm{max}}}{2} + \frac{2\,[\mathrm{P_t}]\cdot[\mathrm{E_t}]}{PLE_{\mathrm{max}}} - [\mathrm{P_t}] - [\mathrm{E_t}]\right)\alpha - K_{\mathrm{P1}} - K_{\mathrm{E1}} \qquad Eq. 4-3c$$

$$FWHM = \log \mathrm{TI}_{50} - \log \mathrm{TF}_{50} \qquad Eq. 4-4$$

$$AUC \cong 1.1 * FWHM * [PLE]_{max} \qquad Eq. 4-5$$

Supporting Information 4-2A. Solving EC_{max}

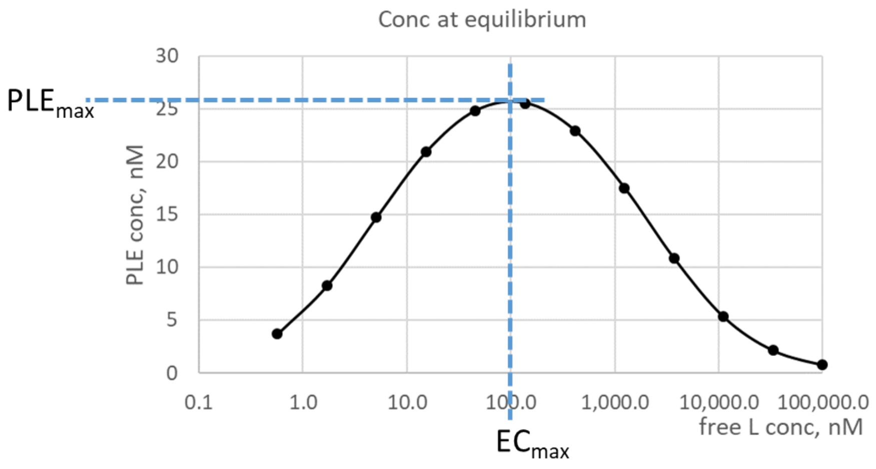

EC_{max} is defined as the concentration of the hetero-bifunctional ligand that gives the maximum concentration of the ternary complex, or the maximum effective concentration. Graphically, this is the concentration that gives the slope of the PLE as a function of L on the semi-log plot zero, or

$$\frac{\mathrm{d}[PLE]}{\mathrm{d}\,log[L]} = 0 \text{ when [L] = } EC_{max}$$

Differentiation of PLE by log[L] can be obtained as below

$$\frac{d[PLE]}{d\log[L]} = \frac{d[PLE]}{d[L]} \cdot \frac{d[L]}{d\ \log[L]} = \frac{\frac{d[PLE]}{d[L]}}{\frac{d\ \log[L]}{d[L]}} = [L]\frac{d[PLE]}{d[L]}$$

Therefore, d[PLE]/dlog[L] = 0 when i) [L] = 0, which is a trivial solution, or when ii) d[PLE]/d[L] = 0. To obtain d[PLE]/d[L], we start with the original function for the [PLE] (Eq. 2-1) as below.

$$[PLE] = \left[f([L]) - \sqrt{f^2([L]) - 4\,[P_t]\cdot[E_t]}\right]/2 \qquad Eq.\ 2-1a$$

$$where\ f([L]) = [P_t] + [E_t] + \frac{1}{\alpha\cdot[L]}([L]+K_{P1})([L]+K_{E1}) \qquad Eq.\ 2-1b$$

$$\frac{d[PLE]}{d[L]} = \left[\frac{df(L)}{d[L]} - \frac{1}{2}\{f^2([L]) - 4\,[P_t]\cdot[E_t]\}^{-\frac{1}{2}}\cdot 2f([L])\frac{df([L])}{d[L]}\right]/2$$

$$= \left[1 - \{f^2([L]) - 4\,[P_t]\cdot[E_t]\}^{-\frac{1}{2}}\cdot f([L])\right]\frac{df([L])}{d[L]}/2 \quad = 0$$

d[PLE]/d[L] = 0 when

iv) $1 - \{f^2([L]) - 4\,[P_t]\cdot[E_t]\}^{-\frac{1}{2}} \cdot f([L]) \;= 0$

or when iii) df([L])/d[L] = 0.

The former case means $f^2([L]) - 4\,[P_t]\cdot[E_t] = f^2([L])$, which is valid only when [L] = ∞. This is the other trivial solution in addition to the first trivial solution of [L] = 0.

The latter case of df([L])/d[L] = 0 is the one that would give the concentration for EC_{max}. To obtain df([L])/d[L], the function f([L]) is expanded as below

$$f([L]) = [P_t] + [E_t] + \frac{1}{\alpha\cdot[L]}([L] + K_{P1})([L] + K_{E1}) \qquad Eq.\ 2-1b$$

$$= [P_t] + [E_t] + \frac{1}{\alpha}([L] + K_{P1} + K_{E1} + \frac{K_{P1}\,K_{E1}}{[L]})$$

$$\frac{df([L])}{d[L]} = \frac{1}{\alpha}\left(1 - \frac{K_{P1}\,K_{E1}}{[L]^2}\right) = 0 \;\; when\ [L]^2 = \ K_{P1}\,K_{E1}$$

Therefore, $[L] \;= \sqrt{K_{P1}\cdot K_{E1}}\;\; when$ df([L])/(d[L]) = 0

In other words,

$$EC_{max} = \sqrt{K_{P1}\cdot K_{E1}} \qquad\qquad Eq.4-1a$$

Equation Eq.4-1b is obtained by taking square of both sides and rearranging them.

$$K_{P1} = \ {EC_{max}}^2\,/K_{E1} \qquad\qquad Eq.4-1b$$

Take log10 of both sides in Eq.4-1a to get

$$\log(\,EC_{max}) = (\log K_{P1} + \log K_{E1})\;/2 \qquad Eq.4-1c$$

Supporting Information 4-2B. Solving for PLE_{max} from EC_{max}

[PLE] = $[PLE]_{max}$ when [L] = EC_{max}

$$[PLE] = \left[f([L]) - \sqrt{f^2([L]) - 4\,[P_t]\cdot[E_t]}\,\right]/2 \qquad Eq.\ 2-1a$$

$$where\ f([L]) = [P_t] + [E_t] + \frac{1}{\alpha\cdot[L]}([L] + K_{P1})([L] + K_{E1}) \qquad Eq.\ 2-1b$$

$$[PLE]_{max} = \left[f(EC_{max}) - \sqrt{f^2(EC_{max}) - 4\,[P_t]\cdot[E_t]}\,\right]/2 \qquad Eq. 4-2a$$

$$f(EC_{max}) = [P_t] + [E_t] + \frac{1}{\alpha\cdot\sqrt{K_{P1}\cdot K_{E1}}}(\sqrt{K_{P1}\cdot K_{E1}} + K_{P1})(\sqrt{K_{P1}\cdot K_{E1}} + K_{E1})$$

$$= [P_t] + [E_t] + \frac{1}{\alpha}\ \left(\sqrt{K_{E1}} + \sqrt{K_{P1}}\right)\left(\sqrt{K_{P1}} + \sqrt{K_{E1}}\right)$$

$$f(EC_{max}) = [P_t] + [E_t] + \frac{1}{\alpha}\ \left(\sqrt{K_{P1}} + \sqrt{K_{E1}}\right)^2 \qquad Eq.\ 4-2b$$

$$= [P_t] + [E_t] + \frac{1}{\alpha}\ \left(K_{P1} + K_{E1} + 2\sqrt{K_{P1}\,K_{E1}}\right)$$

$$= [P_t] + [E_t] + \frac{1}{\alpha}\ (K_{P1} + K_{E1} + 2EC_{max}) \qquad Eq.\ 4-2c$$

Supporting Information 4-2C. Solving EC_{50}

$[PLE] = [PLE]_{max} / 2$ when $[L] = EC_{50}$.

$$PLE(EC_{50}) = \left[f(EC_{50}) - \sqrt{f^2(EC_{50}) - 4\,[P_t]\cdot[E_t]}\,\right]/2 \quad = [PLE]_{max}/2$$

$$f(EC_{50}) - [PLE]_{max} = \sqrt{f^2(EC_{50}) - 4\,[P_t]\cdot[E_t]}$$

$$f^2(EC_{50}) - 2f(EC_{50})\,[PLE]_{max} + [PLE]_{max}{}^2 = f^2([L]) - 4\,[P_t]\cdot[E_t]$$

$$f(EC_{50}) = [PLE]_{max}/2 \;\; + 2/[PLE]_{max} \;\cdot [P_t]\cdot[E_t]$$

$$[P_t] + [E_t] + (EC_{50} + K_{P1})(EC_{50} + K_{E1})/(\alpha\cdot EC_{50})$$
$$= [PLE]_{max}/2 \;\; + 2/[PLE]_{max} \;\cdot [P_t]\cdot[E_t]$$

$$(EC_{50} + K_{P1})(EC_{50} + K_{E1}) = \alpha\cdot EC_{50}\,(\,[PLE]_{max}/2 \;\; + 2/[PLE]_{max} \;\cdot [P_t]\cdot[E_t] \; - [P_t] - [E_t])$$

$$EC_{50}{}^2 - \{\alpha([PLE]_{max}/2 + 2\,[P_t]\cdot[E_t]/[PLE]_{max} - \; [P_t] - [E_t]) \; - \; K_{P1} - K_{E1}\,\}\,EC_{50} + K_{P1}\cdot K_{E1} = 0$$

$$\mathrm{g} = \alpha([PLE]_{max}/2 + 2\,[P_t]\cdot[E_t]/[PLE]_{max} - \; [P_t] - [E_t]) - K_{P1} - K_{E1}$$

$$EC_{50}{}^2 - g \;\cdot EC_{50} + K_{P1}\cdot K_{E1} = 0 \qquad Supp.Eq.4-3$$

$$EC_{50} = \left[g \mp \sqrt{g^2 - 4\;K_{P1}\cdot K_{E1}}\,\right]/2 \qquad Eq.\;4-3a$$

$$\mathrm{g} = \alpha([PLE]_{max}/2 + 2\,[P_t]\cdot[E_t]/[PLE]_{max} - \; [P_t] - [E_t]) - K_{P1} - K_{E1} \qquad Eq.\;4-3c$$

Supporting Information 5-1. Summary of features for Gaussian function and their interconversion to geometric features for ternary complex dose-response curve

Geometric features of the Gaussian function are shown below along with their translation to the geometric features of the ternary complex dose-response curve shown in **Supporting Information 4-1**. Derivation for the equation Eq.5-7 is shown in **Supporting Information 5-2**.

$$\text{Gaussian} = \text{Amplitude} * \exp^{-0.5\left(\frac{X-\text{Mean}}{\text{SD}}\right)^2}$$

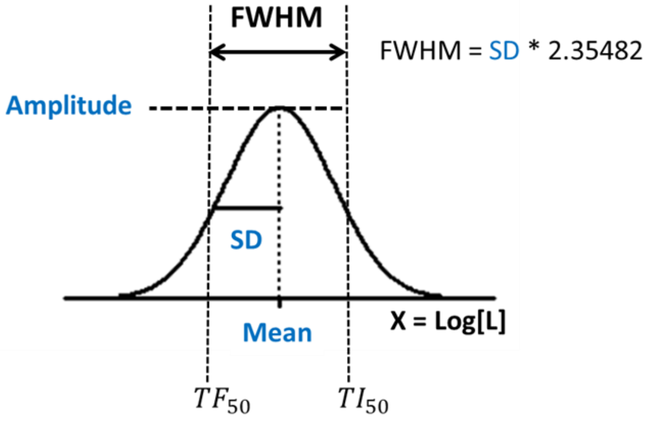

$$\text{TCS} = \text{Amplitude} * \exp^{-0.5\left(\frac{\log[L]-\text{Mean}}{\text{SD}}\right)^2} \qquad Eq.5-1$$

$$\text{Amplitude} = TCS_{max} \qquad Eq.5-2$$

$$\text{Mean} = \log(EC_{max}) = \log\sqrt{K_{P1} \cdot K_{E1}} \qquad Eq.5-3$$

$$\text{SD} = (\log TI_{50} - \log TF_{50}) / 2.35482 \qquad Eq.5-4$$

$$EC_{max} = 10^{\text{Mean}} \qquad Eq.5-5$$

$$TF_{50} = 10^{\text{Mean} - 1.17741\ \text{SD}} \qquad Eq.5-6a$$

$$TI_{50} = 10^{\text{Mean} + 1.17741\ \text{SD}} \qquad Eq.5-6b$$

$$\log EC_X = \text{Mean} \mp \sqrt{2\ln\left(\frac{100}{X}\right)}\ \text{SD} \qquad Eq.5-7$$

$$\log TF_X = \text{Mean} - \sqrt{2\ln\left(\frac{100}{X}\right)}\ \text{SD} \qquad Eq.5-7a$$

$$\log TI_X = \text{Mean} + \sqrt{2\ln\left(\frac{100}{X}\right)}\ \text{SD} \qquad Eq.5-7b$$

$$AUC_{TCS} = 2.50663\ \text{Amplitude} * \text{SD} \qquad Eq.5-8$$

Supporting Information 5-2. EC_X for concentration that gives X % of maximal ternary complex formation

$$TCS = Amplitude * exp^{-0.5(\frac{\log[L]-Mean}{SD})^2} \qquad Eq. 5-1$$

$$\log EC_X = Mean \mp \sqrt{2\ln\left(\frac{100}{X}\right)}\ SD \qquad Eq. 5-7$$

At EC_X, TCS/Amplitude = X/100 or TCS = Amplitude * X/100

$$TCS = Amplitude * exp^{-0.5(\frac{\log[L]-Mean}{SD})^2} = \frac{X}{100} * Amplitude \qquad \text{when } L = EC_X$$

$$exp^{-0.5(\frac{\log[L]-Mean}{SD})^2} = \frac{X}{100}$$

$$-0.5\left(\frac{\log[L]-Mean}{SD}\right)^2 = \ln\left(\frac{X}{100}\right)$$

$$\left(\frac{\log[L]-Mean}{SD}\right)^2 = 2\ln\left(\frac{100}{X}\right)$$

$$\frac{\log[L]-Mean}{SD} = \mp\sqrt{2\ln\left(\frac{100}{X}\right)}$$

$$\log[L] = Mean \mp SD\sqrt{2\ln\left(\frac{100}{X}\right)} \qquad \text{when } [L] = EC_X$$

Supporting Information 5-3. Iterative LeastSumSquare method (iLSS) for Gaussian curve fitting of the experimental dose-response data using total ligand concentration

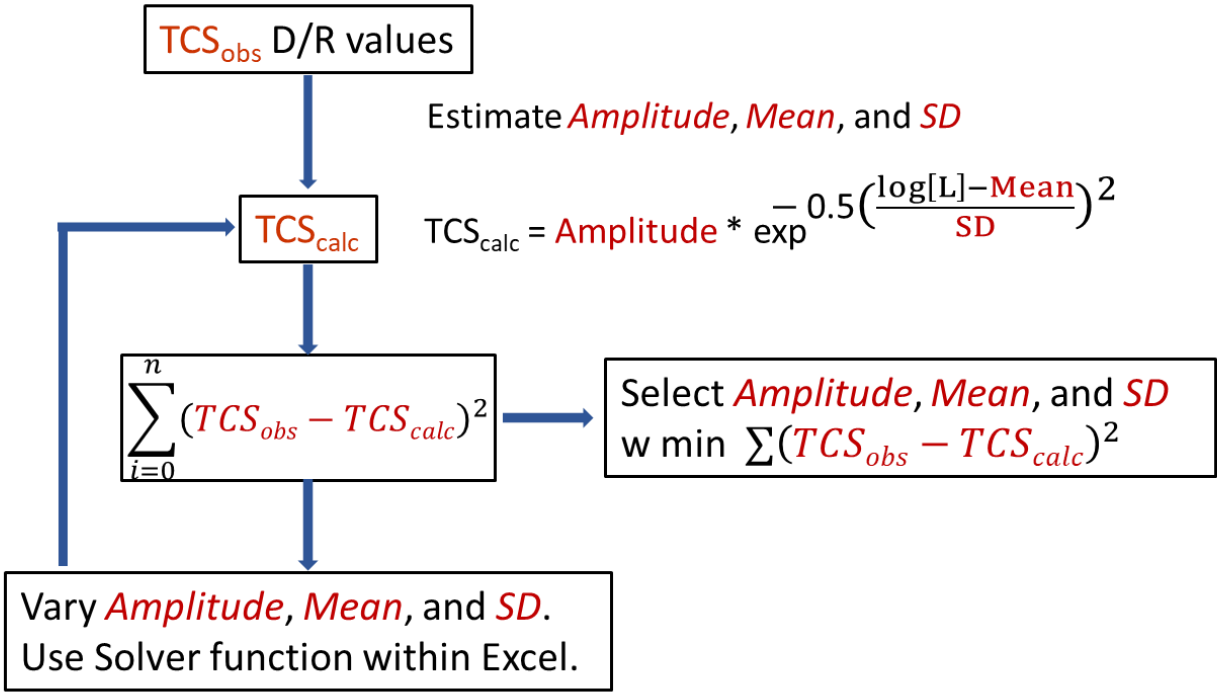

The iterative LeastSumSquare (iLSS) method was used for curve fitting of the simulated ternary complex signal (TCS) dose-response data with Gaussian function. Log10 values were used for all concentration values for this function. Initial set of values were assigned to each of the three parameters for the function, Amplitude, Mean, and SD using a simple rule as follows: maximum observed TCS signal was assigned to Amplitude. Concentration that gave the maximum TCS signal was assigned to Mean. Standard deviation of all concentration values in log10 base was assigned to SD. With this initial set of values, expected TCS values were calculated and compared with the measured TCS values. Sum of the square of the differences at each ligand concentration (SumSquare) was calculated. Small variations were tested for all parameters in a systematic manner and the set of parameters that gives the minimum value of SumSquare (LeastSumSquare) was searched. In other words, the parameters were optimized to minimize the SumSquare value. Multiple iterations are required for this method and this process was automated by using Solver function within Microsoft Excel. For successful optimization of the parameters for this method, starting with a reasonable set of initial test values is important. The rule described above worked satisfactorily in most cases. A template program is provided under "**Gss_totalL**" tab in an Excel file, **BHan_TCextLSS_v3.5.4_200221.xlsx**. Solver function within Microsoft Excel needs to be enabled by following the menu options under File → Options → Add-Ins → Analysis ToolPak → then checking Solver Add-In option. Once enabled, the Solver function can be accessed under Data → Solver. The program automatically calculates not only the three parameters of the Gaussian function but also other useful parameters such as FWHM and AUC.

Supporting Information 6A. Iterative LeastSumSquare method (iLSS) for optimizing equilibrium constants (K_{E1}, K_{P1}, and α) in the PLE function from experimental dose-response data using total ligand concentration

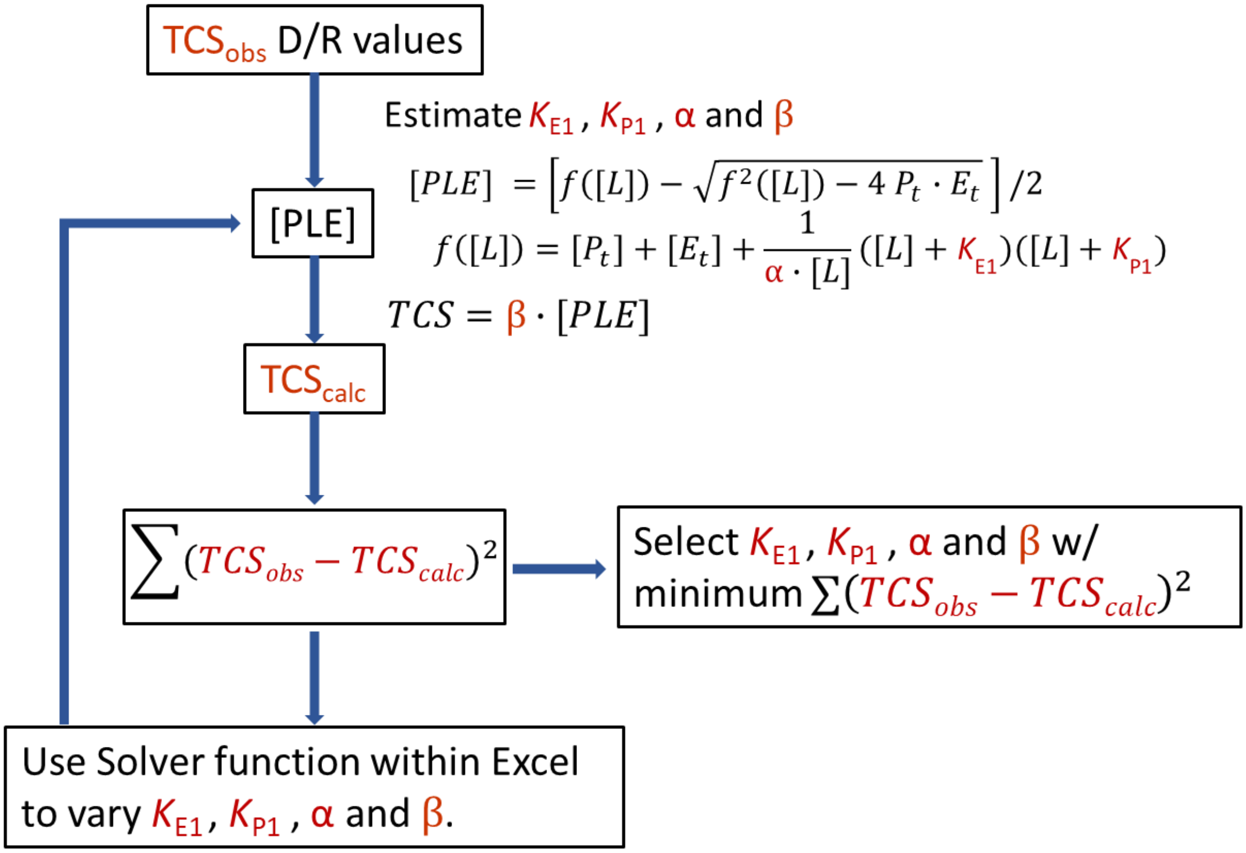

$$[PLE] = \left[f([L]) - \sqrt{f^2([L]) - 4\,P_t \cdot E_t} \right] / 2$$

$$f([L]) = [P_t] + [E_t] + \frac{1}{\alpha \cdot [L]}([L] + K_{E1})([L] + K_{P1})$$

$$TCS = \beta \cdot [PLE]$$

$$\sum (TCS_{obs} - TCS_{calc})^2$$

The same iLSS method shown in Supporting Information 5A can be used to curve fit the experimental dose-response data with the PLE function and obtain optimized values for the equilibrium constants K_{P1}, K_{E1}, α, and the system conversion factor β. In this case, unmodified total ligand concentration should be used as opposed to the log10 value for the Gaussian function. However, initial evaluation of the method using the simulated experimental data showed that the equilibrium constants from this exercise deviated significantly from the true values due to ligand depletion. Information on free ligand concentration was needed for this purpose. A numeric approach can be used to get a highly precise value for the free ligand concentration as explained in the **Supporting Information 6B**. Extended LeastSumSquare (extLSS) method (**Supporting Information 6C**) was developed to incorporate numeric calculation of the free ligand concentrations in every iteration within the iLSS method.

Supporting Information 6B. Free ligand concentration in Ternary System

The concentration symbol, [ ], is omitted for [L], [PLE], [P_t], and [E_t] below for convenience.

$$L_t = L + PL + EL + PLE$$

$$L_t = L + (P_t - PLE)\frac{L}{L + K_{P1}} + (E_t - PLE)\frac{L}{L + K_{E1}} + PLE$$

$$(L_t - PLE)(L + K_{P1})(L + K_{E1}) = L(L + K_{P1})(L + K_{E1}) + (P_t - PLE)L(L + K_{E1})(E_t - PLE)L(L + K_{P1})$$

$$\boldsymbol{L}^3 + (P_t + E_t + K_{P1} + K_{E1} - L_t - PLE)\boldsymbol{L}^2 + (P_tK_{E1} + E_tK_{P1} + K_{P1}K_{E1} - L_tK_{P1} - L_tK_{E1})\boldsymbol{L} + K_{P1}K_{E1}(PLE - L_t) = 0$$

Solution for a cubic function, y = $L^3 + aL^2 + bL + c$ can be obtained numerically as illustrated in the graph below:

Starting with an initial value of $L_0 = L_t$, find a line that passes through (L_0, y_0) with a slope of $y' = 3L^2 + 2aL + b$. Intercept of this line at the horizontal axis is (L_1, 0). Repeat the above process until y value approaches zero. Usually, 5 cycles (L_5) is more than sufficient to get an excellent estimate of L that satisfies the equation $y = L^3 + aL^2 + bL + c = 0$.

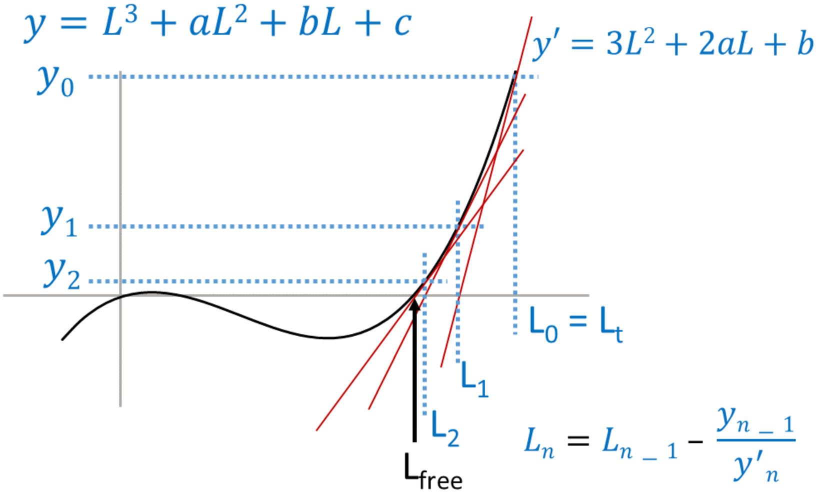

$$L^3 + aL^2 + bL + c = 0$$

$$a = P_t + E_t + K_{P1} + K_{E1} - L_t - PLE$$

$$b = P_t K_{E1} + E_t K_{P1} + K_{P1}K_{E1} - L_t K_{P1} - L_tK_{E1}$$

$$c = K_{P1}K_{E1}(PLE - L_t)$$

$$L_n = L_{n\,\_\,1} - \frac{y_{n\,\_\,1}}{y'_n}$$

$$L_{free} = L \text{ when } y(L) = L^3 + aL^2 + bL + c \approx 0$$

Supporting Information 6C. Extended LeastSumSquare (extLSS) method for finding equilibrium constants from Ternary Complex Dose-Response data

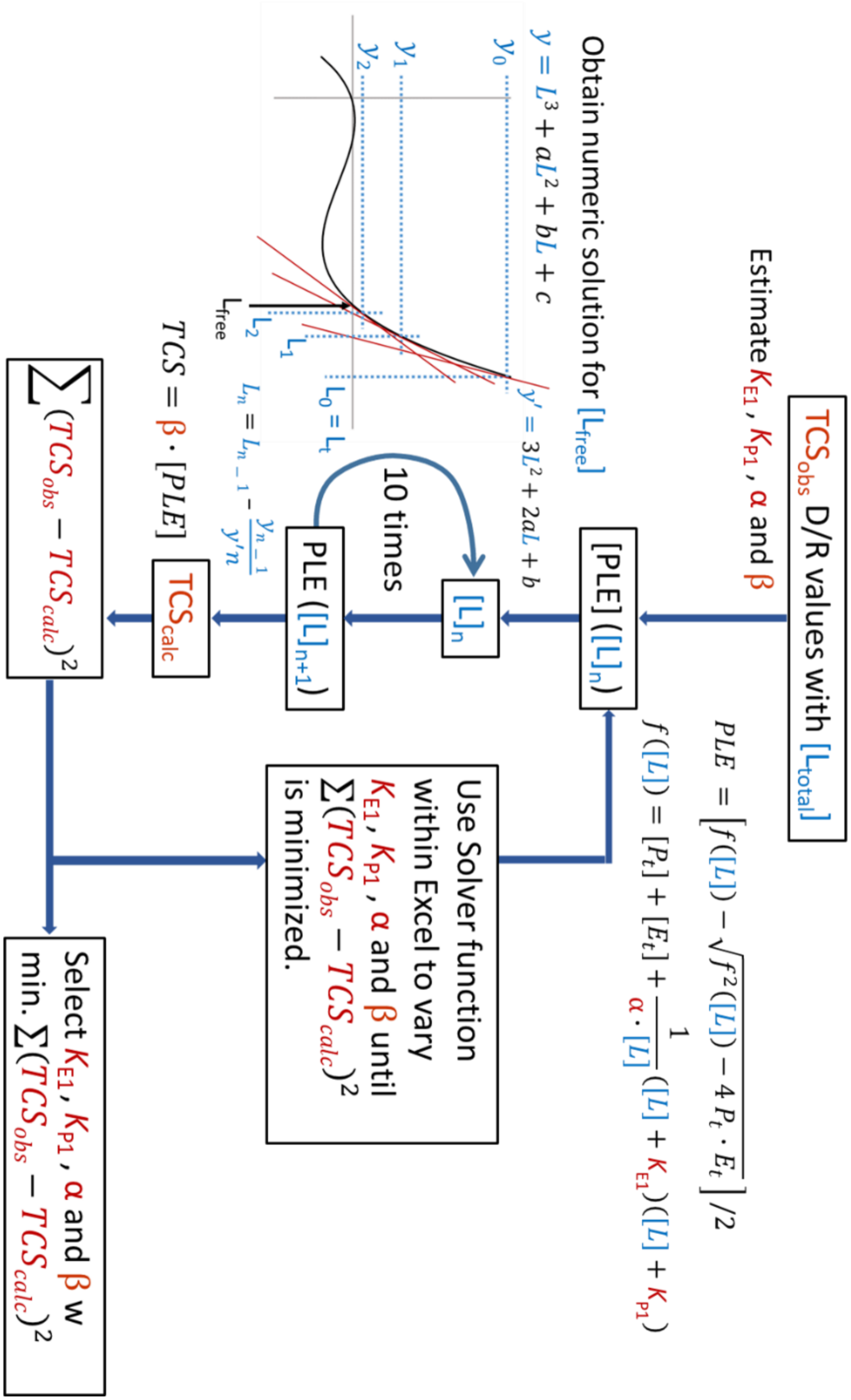

In the extended LeastSumSquare (extLSS) method, numeric calculation of the free ligand concentration through an iterative process was looped into the iLSS method for every iteration of the parameter adjustment. Similar to the Gaussian curve fitting of the data with iLSS method, selection of reasonable initial condition was important for successful estimation of the equilibrium constants. EC_{max} and TCS_{max} (maximum ternary complex signal) values from the Gaussian curve fitting of the same data using the total ligand concentration were used to generate initial values for the four parameters, K_{P1}, K_{E1}, α, and β in combination with the information on total protein concentration. Initial value for K_{E1} was assigned to be 100 nM, and the initial value for K_{P1} was calculated from the relationship $K_{P1} = EC_{max}^2/K_{E1}$. Median value of $[PLE]_{max}$ across all data sets was assumed to be 10% of the total protein concentration and the initial value for β for individual data set was calculated from the tentative $[PLE]_{max}$ value and the measured TCS_{max} value. With these assumptions, initial value for α was calculated from the Eq.6-1a in **Supporting Information 6D**. When system conversion factor β was floated for initial evaluation and for system calibration, it was obtained from $TCS_{max} = \beta * [PLE]_{max}$. Otherwise, β was fixed to the calibrated value. A template program is provided under "**extLSS_freeL**" tab in the Excel file **BHan_TCextLSS_v3.5.4_200221.xlsx**. Solver function needs to be enabled within Microsoft Excel as explained for the iLSS method in the **Supporting Information 5-3**. Evaluation of the extLSS method using simulated training data set is described in the **Supporting Information 6E**.

The Excel-based curve fitting programs, **BHan_TCextLSS_v3.5.4_200221.xlsx**, contains

1. **Gss_ totalL**; Gaussian Curve fitting on total [L] by iterative LeastSumSquare (iLSS) method
2. **extLSS_freeL**; Gaussian Curve fitting on total [L] by iLSS method + PLE curve fitting on free [L] by extLSS method
3. **Instruction**; instruction on how to use the program

User-provided dose-response data and total protein concentration information are entered within the areas highlighted with bright yellow. Back up copy of each module is placed as "Hidden" tabs. If restoration of default settings is desired or if the program codes get corrupted within the program areas, them make a copy of the desired area from the backup tab and paste into the affected areas.

Contact Bomie Han at bomiehan@lilly.com for further questions/instructions.

Supporting Information 6C. Equations for α, K_{P1}, & K_{E1}

$$\alpha = \frac{[PLE]_{max}\left(\sqrt{K_{P1}} + \sqrt{K_{E1}}\right)^2}{([P_t] - [PLE]_{max})([E_t] - [PLE]_{max})} = \frac{[PLE]_{max}(K_{P1} + K_{E1} + 2EC_{max})}{([P_t] - [PLE]_{max})([E_t] - [PLE]_{max})} \qquad Eq. 6-1a$$

$$= \frac{2(EC_{50} + K_{P1})(EC_{50} + K_{E1})\,[PLE]_{max}}{(2[P_t] - [PLE]_{max})(2[E_t] - [PLE]_{max})EC_{50}} \qquad Eq. 6-1b$$

$$K_{P1} = \left[h \pm \sqrt{h^2 - 4{EC_{max}}^2}\,\right]/2 \qquad Eq. 6-2a$$

$$K_{E1} = \left[h \mp \sqrt{h^2 - 4{EC_{max}}^2}\,\right]/2 \qquad Eq. 6-2b$$

$$h = \alpha\frac{([P_t] - [PLE]_{max})([E_t] - [PLE]_{max})}{[PLE]_{max}} - 2EC_{max} \qquad Eq. 6-2c$$

Supporting Information 6D-1. Solving for α from $[PLE]_{max}$ or from EC_{50}

Solving α from $[PLE]_{max}$

$$[PLE]_{max} = \left[f(EC_{max}) - \sqrt{f^2(EC_{max}) - 4\,[P_t]\cdot[E_t]}\,\right]/2 \qquad Eq. 4-2a$$

$$f(EC_{max}) = [P_t] + [E_t] + \frac{1}{\alpha}\ \left(\sqrt{K_{P1}} + \sqrt{K_{E1}}\right)^2 \qquad Eq.\ 4-2b$$

$$2\cdot[PLE]_{max} - f(EC_{max}) = -\sqrt{f^2(EC_{max}) - 4\,[P_t]\cdot[E_t]}$$

$$4\cdot{[PLE]_{max}}^2 - 4\cdot[PLE]_{max}\cdot f(EC_{max}) + f^2(EC_{max}) \ = f^2(EC_{max}) - 4\,[P_t]\cdot[E_t]$$

$${[PLE]_{max}}^2 - [PLE]_{max}\cdot f(EC_{max}) + \ [P_t]\cdot[E_t] = 0$$

$$\left({PLE_{max}}^2 + \ [P_t]\cdot[E_t]\ \right)/[PLE]_{max} = f(EC_{max}) = [P_t] + [E_t] + \frac{1}{\alpha}\ \left(\sqrt{K_{P1}} + \sqrt{K_{E1}}\right)^2$$

$$\left({[PLE]_{max}}^2 + \ [P_t]\cdot[E_t]\ \right)/[PLE]_{max} - [P_t] - [E_t] = \frac{1}{\alpha}\ \left(\sqrt{K_{P1}} + \sqrt{K_{E1}}\right)^2$$

$$\{{[PLE]_{max}}^2 + \ [P_t]\cdot[E_t]\ - [PLE]_{max}\ ([P_t] + [E_t])\}/[PLE]_{max} = \frac{1}{\alpha}\ \left(\sqrt{K_{P1}} + \sqrt{K_{E1}}\right)^2$$

$$\frac{([PLE]_{max} - [P_t])([PLE]_{max} - [E_t])}{[PLE]_{max}} = \frac{1}{\alpha}\ \left(\sqrt{K_{P1}} + \sqrt{K_{E1}}\right)^2$$

$$\alpha = \frac{[PLE]_{max}\left(\sqrt{K_{P1}} + \sqrt{K_{E1}}\right)^2}{([P_t]\ - [PLE]_{max})([E_t]\ - [PLE]_{max})} = \frac{[PLE]_{max}(K_{P1} + K_{E1} + 2EC_{max})}{([P_t]\ - [PLE]_{max})([E_t]\ - [PLE]_{max})} \qquad Eq. 6-1a$$

Solving for α from EC_{50}

$$g = \alpha([PLE]_{max}/2 + 2\,[P_t] \cdot [E_t]/[PLE]_{max} - [P_t] - [E_t]) - K_{P1} - K_{E1} \qquad Eq.\ 4-3c$$

$$EC_{50}{}^2 - g \cdot EC_{50} + K_{P1} \cdot K_{E1} = 0 \qquad Supp.Eq.4-3$$

$$(EC_{50}{}^2 + K_{P1} \cdot K_{E1})/EC_{50} = \alpha([PLE]_{max}/2 + 2\,[P_t] \cdot [E_t]/[PLE]_{max} - [P_t] - [E_t]) - K_{P1} - K_{E1}$$

$$\{EC_{50}{}^2 + K_{P1} \cdot K_{E1} + (K_{P1} + K_{E1})EC_{50}\}/EC_{50}$$
$$= \alpha([PLE]_{max}/2 + 2\,[P_t] \cdot [E_t]/[PLE]_{max} - [P_t] - [E_t])$$

$$(EC_{50} + K_{P1})(EC_{50} + K_{E1})/EC_{50} = \alpha([PLE]_{max} + 4\,[P_t] \cdot [E_t]/[PLE]_{max} - 2[P_t] - 2[E_t])\,/2$$

$$\frac{[PLE]_{max}(EC_{50} + K_{P1})(EC_{50} + K_{E1})}{EC_{50}} = \alpha\{[PLE]_{max}{}^2 + 4\,[P_t] \cdot [E_t] - 2([P_t] + [E_t])[PLE]_{max}\}/2$$

$$\frac{2\,[PLE]_{max}(EC_{50} + K_{P1})(EC_{50} + K_{E1})}{EC_{50}} = \alpha(2[P_t] - [PLE]_{max})(2[E_t] - [PLE]_{max})$$

$$\alpha = \frac{2(EC_{50} + K_{P1})(EC_{50} + K_{E1})\,[PLE]_{max}}{(2[P_t] - [PLE]_{max})(2[E_t] - [PLE]_{max})EC_{50}} \qquad Eq.6-1b$$

Supporting Information 6D-2. Solving K_{P1} & K_{E1}

$$\alpha = \frac{[PLE]_{max}(K_{P1} + K_{E1} + 2EC_{max})}{([P_t] - [PLE]_{max})([E_t] - [PLE]_{max})} \qquad Eq. 6-1a$$

$$\alpha\,([P_t] - [PLE]_{max})([E_t] - [PLE]_{max}) = [PLE]_{max}(K_{P1} + K_{E1} + 2EC_{max})$$

$$K_{P1} + K_{E1} = \alpha\frac{([P_t] - [PLE]_{max})([E_t] - [PLE]_{max})}{[PLE]_{max}} - 2EC_{max}$$

$$K_{P1} + {EC_{max}}^2 / K_{P1} = \alpha\frac{([P_t] - [PLE]_{max})([E_t] - [PLE]_{max})}{[PLE]_{max}} - 2EC_{max}$$

$${K_{P1}}^2 - \left(\alpha\frac{([P_t] - [PLE]_{max})([E_t] - [PLE]_{max})}{[PLE]_{max}} - 2EC_{max}\right)K_{P1} + {EC_{max}}^2 = 0$$

$$K_{P1} = \left[h \pm \sqrt{h^2 - 4{EC_{max}}^2}\,\right]/2 \qquad Eq. 6-2a$$

$$K_{E1} = \left[h \mp \sqrt{h^2 - 4{EC_{max}}^2}\,\right]/2 \qquad Eq. 6-2b$$

$$h = \alpha\frac{([P_t] - [PLE]_{max})([E_t] - [PLE]_{max})}{[PLE]_{max}} - 2EC_{max} \qquad Eq. 6-2c$$

Supporting Information 6E. Equilibrium constants determined by the extended LeastSumSquare (extLSS) method from the simulated Ternary Complex Dose-Response data

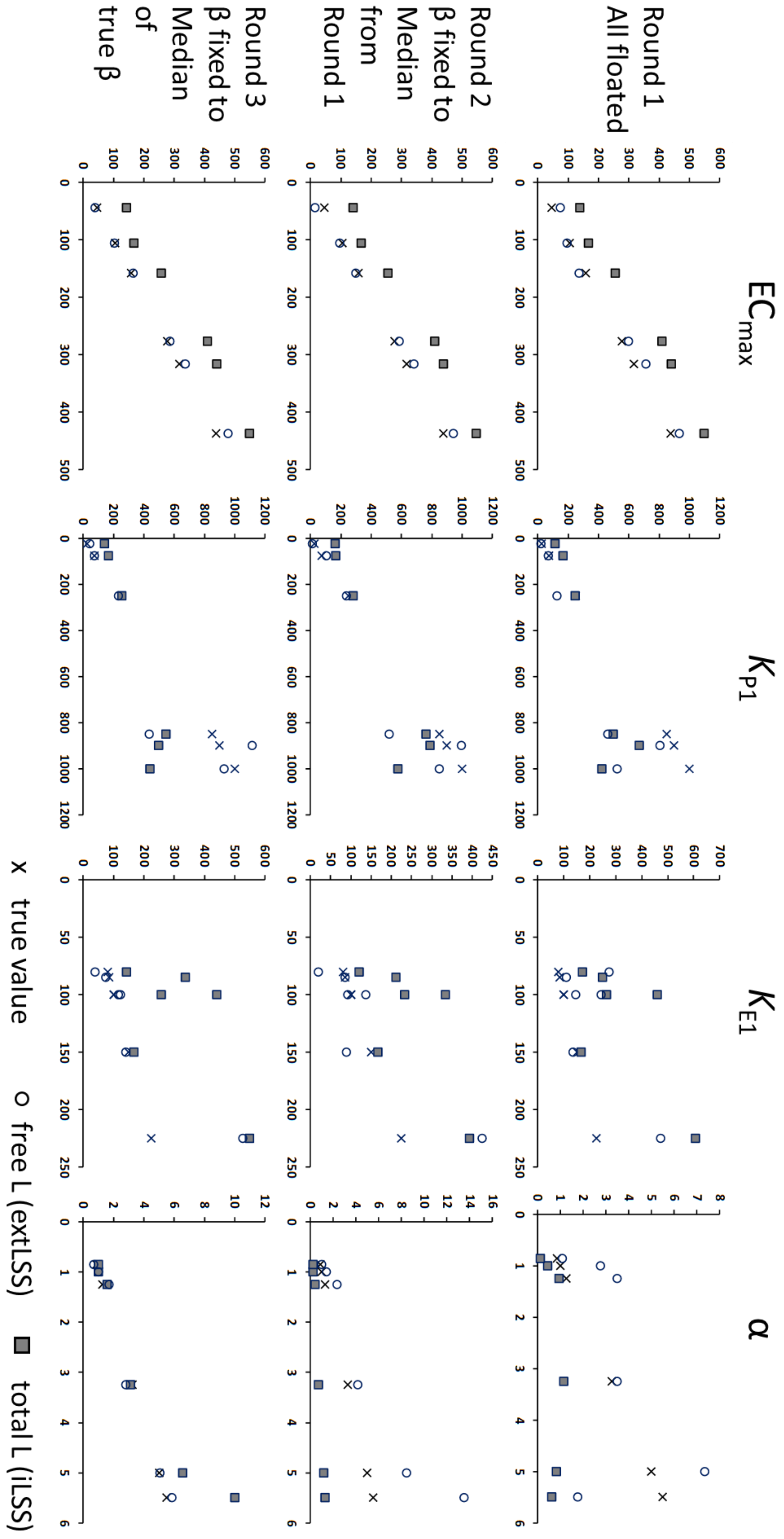

Extended LeastSumSquare method (extLSS) shown schematically in **Supporting Information 6C** illustrates how the equilibrium constants (K_{P1}, K_{E1}, and α) as well as the system conversion factor (β) can be obtained by iterative optimization of the parameters to fit the experimental data. This method combines an iterative LeastSumSquare (iLSS) method described in **Supporting Information 6A** with a numeric method of calculating free ligand concentrations in the ternary complex system as described in **Supporting Information 6B**. Equations given in the **Supporting Information 6D** were used to generate initial test values for parameter optimization by extLSS method. An Excel-based template program for the extLSS method is available as a separate file, **BHan_TCextLSS_v3.5.4_200221.xlsx**. Outcomes from three rounds of optimization exercise are compared in this graph: In the first round (***Top*** panel), all four parameters (K_{P1}, K_{E1}, α, and β) were floated. In the second round (***Middle*** panel), β was fixed to the median of β values for all data sets from the first round. In the third round (***Bottom*** panel), β was fixed to the median of true β values for all data sets, representing a system that has been correctly calibrated. Optimized values for EC_{max}, K_{P1}, K_{E1}, and α are compared among true values (cross hairs), extLSS method (open circles), and iLSS method (filled circles) in each case.

In all cases, the optimized value of EC_{max} was very close to the true value with the extLSS method, whereas iLSS method consistently reported higher value than the true value. This was consistent with lack of consideration for ligand depletion in the iLSS method based on total ligand concentration. Assigning individual values for K_{P1} and K_{E1} from this optimization exercise was more challenging when the simulated data contained random errors. Overall, for ligands with similar K_{E1} values, the extLSS method performed very well to predict changes in the K_{P1} or the binary affinity for the target protein, but the system performed poorly in correctly predicting changes in K_{E1} value in all cases. This behavior stems from the symmetry in mathematical terms between the target protein P and the E3 ligase E. When either K_{P1} or K_{E1} values are significantly different from the initial test values for the optimization iteration, then the optimization method cannot determine which one needs to be changed to get the best fit. Some of the optimized values in this figure have the K_{P1} and K_{E1} values flipped. Optimized binary equilibrium constants (K_{P1} and K_{E1}) will have to be compared to structural information of the hetero-bifunctional ligand for proper assignment of the K_{P1} and K_{E1} values. Optimizing the cooperativity factor α from the extLSS method did not perform well when all four parameters were floated (***Top*** panel). When the calibration factor β was fixed to the median value from the first round of optimization (***Middle*** panel), the rank order of the α values was correctly predicted even though the absolute values showed deviations from the true values especially for high α values. When the β value was fixed to the true median value (***Bottom*** panel), the optimized α values showed an excellent agreement with the true values. In other words, when the system is correctly calibrated to obtain the best β value, then the extLSS method can correctly give an excellent estimates of the cooperativity factor α values.

From these observations, I suggest the following steps for best estimation of the equilibrium parameters from the ternary complex dose-response data. Once the assay system is chosen for measurement of the ternary complex, evaluate existing hetero-bifunctional ligands using simple Gaussian curves and determine EC_{max} and TCS_{max} values based on total ligand concentration. Choose multiple ligands with various EC_{max} and TCS_{max} values as a set of calibrators for the system. When significant changes in affinity for the E3 ligase is expected among different ligands based on structural information, then select a separate set of ligands with similar expected affinity for the E3 ligase within the set. Use these set of

calibrators to go through extLSS method with all four parameters floating. Alternatively, the system can be calibrated using a ligand(s) with known equilibrium constants, if available. Next step is to calculate the median value of the system calibration factor β from these calibrators and the system is considered "calibrated." Use the calibrated value of β for all subsequent optimization of the three equilibrium constants, K_{P1}, K_{E1}, and α, for these and other ligands. As with all non-linear curve fitting methods that rely on minimizing the sum of squares of the difference between the measured and predicted values, successful search for optimum set of parameters depend on starting with a reasonable set of initial test values. In this extLSS method in the template provided in the **BHan_TCextLSS_v3.5.4_200221.xlsx**, outcomes from the Gaussian curve fitting of the experimental data with the total ligand concentration were used to generate a "reasonable" set of initial test values.

Once the system conversion factor β is determined from the curve fitting, then the measured TCS data can be converted to molar concentration of the ternary complex PLE. For this reason, β can be also called a system calibration factor. When the system is calibrated, it becomes possible to calculate what fraction of the total target protein was engaged in the ternary complex under given experimental condition. It may seem counterintuitive at first that absolute concentrations can be calculated from relative signal intensities without an external calibration. The reason why this is possible is because the PLE function (Eq. 2-1a/b) contains reference to known molar concentrations of the total protein, $[P_t]$ and $[E_t]$, which serve as a frame of reference in this calculation.

Supporting Information 7. Summary

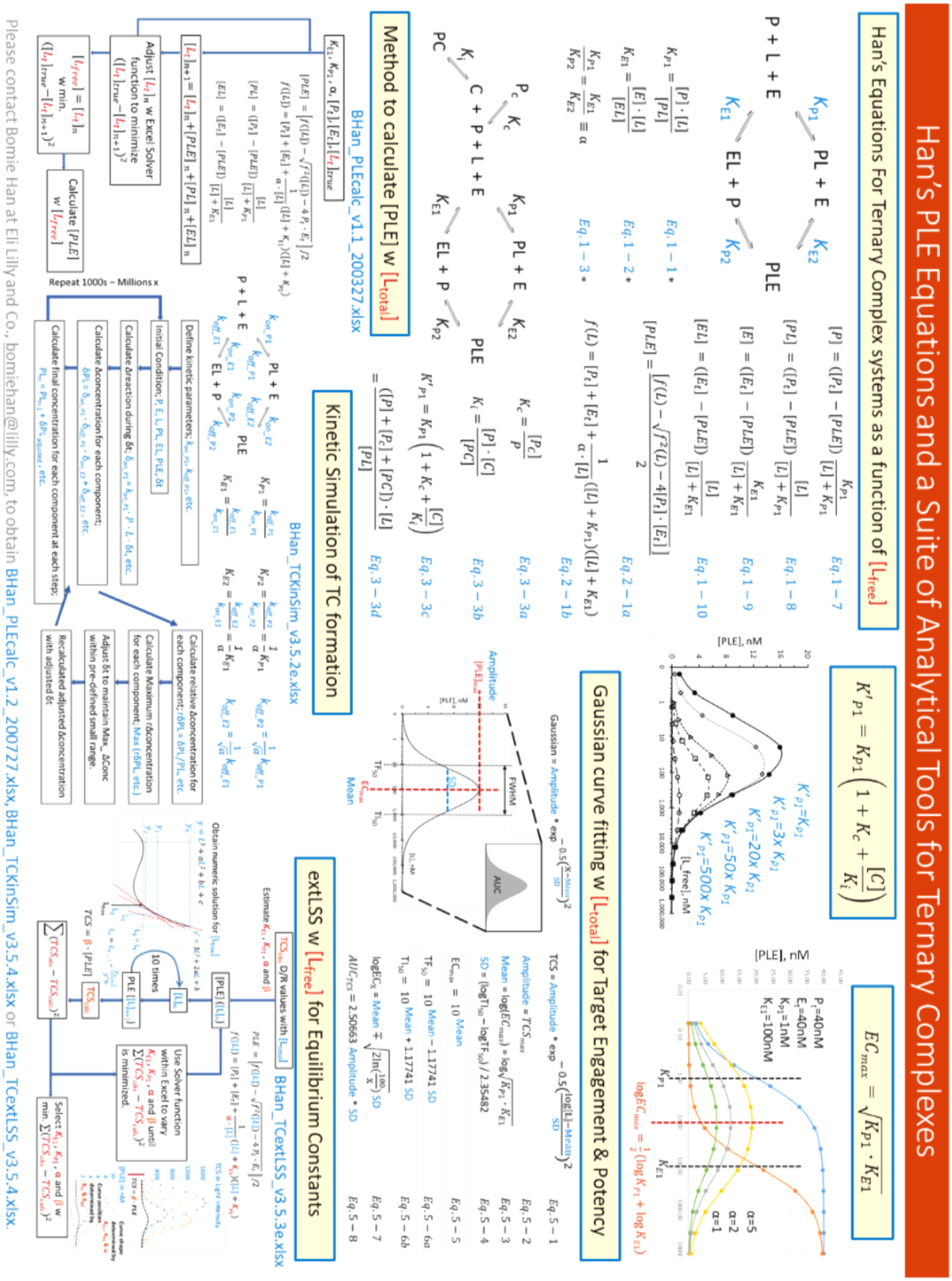

Supplement: Supporting Information [file supp_RA120.014715_161351_2_supp_587124_qfn7xg.pdf]
